# Mapping sequences by parts

**DOI:** 10.1186/1748-7188-2-11

**Published:** 2007-09-19

**Authors:** Gilles Didier, Carito Guziolowski

**Affiliations:** 1 Institut de Mathématiques de Luminy, 163 avenue de Luminy, Case 907, 13288 Marseille Cedex 9, France.; 2 Projet Symbiose, IRISA – campus de Beaulieu, 35042 Rennes Cedex, France.

## Abstract

**Background:**

We present the *N*-map method, a pairwise and asymmetrical approach which allows us to compare sequences by taking into account evolutionary events that produce shuffled, reversed or repeated elements. Basically, the optimal *N*-map of a sequence *s *over a sequence *t *is the best way of partitioning the first sequence into *N *parts and placing them, possibly complementary reversed, over the second sequence in order to maximize the sum of their gapless alignment scores.

**Results:**

We introduce an algorithm computing an optimal *N*-map with time complexity *O *(|*s*| × |*t*| × *N*) using *O *(|*s*| × |*t*| × *N*) memory space. Among all the numbers of parts taken in a reasonable range, we select the value *N *for which the optimal *N*-map has the most significant score. To evaluate this significance, we study the empirical distributions of the scores of optimal *N*-maps and show that they can be approximated by normal distributions with a reasonable accuracy. We test the functionality of the approach over random sequences on which we apply artificial evolutionary events.

**Practical Application:**

The method is illustrated with four case studies of pairs of sequences involving non-standard evolutionary events.

## Background

Classic alignments methods are unable to extract homologies involving shuffled, reverse-complemented or repeated elements between sequences, despite the fact that there are identified mechanisms of evolution of sequences which lead to such types of homologies. This can happen on large scale with genome rearrangements but it can also occur on a smaller scale, for instance within genes, with domain recombinations, duplications, exon shufflings, *etc*.

On the other hand, there are few methods allowing us to compare sequences with relaxed assumptions about conservation of linear order and one-to-one association of positions between sequences [1-3]. In particular, as it is pairwise and asymmetrical, the approach proposed in [1] is similar to the work presented here. The authors introduce the transformation distance, similar to the Levenstein distance between sequences, which includes editing operations like transposition, duplication, *etc*. The algorithmic complexity of the computation of this distance, which was initially high, has been improved in [4].

However, the transformation distance has some drawbacks; mainly, it does not take into account mutations. In [2], the authors introduce the Glocal alignment method which allows one to compare sequences with shuffled or inverted elements. The main idea of their work is to combine local and global alignments. During the first stage, the method selects conserved segments (local) and during the second stage, it chains an optimal subset of the pairs of segments previously selected (global). Many specialized approaches have been developed to model the specific evolutions "by blocks" of certain elements of sequences: minisatellites [5] or swaps in proteins sequences [6]. In the latter, the method is based on selection of common segments by local alignments scores and can be applied in a more general framework. The approach proposed in [3], mostly applied to more than two sequences, proceeds in similar manner in its first stage, then performs post-treatments and a graph representation of common elements of sequences.

For simplicity, we present the *N*-maps without taking into account inversions. Some hints will be given about how to extend the definitions and the algorithms in order to handle this type of evolution. Under this restriction, the (optimal) *N*-map of a sequence *s *over a sequence *t *is basically the way of cutting *s *into *N *parts that maximizes the sums of the scores of the gapless alignments of all the *N *parts against *t*. The gapless alignments can be local or global and so can be the *N*-map. This approach can be seen as a generalization of the "alignment with a fixed number of gaps" method initially introduced in [7] and recently studied in [8,9]. As this method, our approach is an attempt to avoid the introduction of some arbitrary costs on the transformations between sequences (like gap penalties in the case of alignment). For this purpose, we need a concrete way to determine the "best" number *N *of parts for mapping a sequence *s *over a sequence *t*. As in [9], we define this problem from a probabilistic point of view. Practically, we choose the number of parts leading to the most significant optimal score. The significance is empirically evaluated among pairs of independent identically distributed (iid) random sequences of same lengths and symbols distributions as *s *and *t*.

The rest of this paper is organized as follows. Section 1 is devoted to formal definitions and basic properties of the *N*-maps. We present the algorithms computing the optimal scores and correponding *N*-maps of a sequence *s *over a sequence *t *in Section 2. The algorithmic complexities of these computations are *O *(|*s*| × |*t*| × *N*) in time and *O *(|*s*| + |*t*| × *N*) in memory space. These complexities have an extra factor *N *with regard to the classical pairwise alignment algorithms. However typical values of interest of *N *are small compared to the lengths of the sequences: choosing a number of parts of the same order as the lengths of the sequences does not make any sense. The choice of the number of parts is discussed in Section 3, in which we investigate the distributions of the scores of the optimal *N*-maps of random sequences. In particular, empirical evidences lead us to approximate these distributions by normal ones and to measure the significance of optimal scores in terms of *Z*-values. The approach is evaluated in Section 4 by applying artificial evolutionary events over random sequences and by measuring the ability of the approach to retrieve the corresponding homologous segments. Section 5 shows four case studies of sequences (two pairs of proteins, a pair of DNA sequences of transposon elements and a pair of sequences of genes of microbial genomes) in which the homologies cannot be reported by a classic alignment. Finally in Section 6, we discuss the approach and present some research directions we plan to explore.

The sources of the software computing *N*-maps are available at [10]. We also provide additional utilities to estimate *Z*-values, represent *N*-maps as pictures (see Section 5), filter, merge and extract common segments.

## 1 Notations and Definitions

We consider sequences (or strings) over some finite alphabet A
 MathType@MTEF@5@5@+=feaafiart1ev1aaatCvAUfKttLearuWrP9MDH5MBPbIqV92AaeXatLxBI9gBaebbnrfifHhDYfgasaacH8akY=wiFfYdH8Gipec8Eeeu0xXdbba9frFj0=OqFfea0dXdd9vqai=hGuQ8kuc9pgc9s8qqaq=dirpe0xb9q8qiLsFr0=vr0=vr0dc8meaabaqaciaacaGaaeqabaqabeGadaaakeaat0uy0HwzTfgDPnwy1egaryqtHrhAL1wy0L2yHvdaiqaacqWFaeFqaaa@3820@ of elements called letters or symbols. In practical applications, symbols can represent nucleotides, amino acids or genes. The elements of a sequence *s *are indexed from 1 to |*s*|, where |*s*| denotes the length of *s*, *i.e*. *s *= *s*_1 _*s*_2 _... *s*_|*s*|_. For 1 ≤ *i *≤ *j *≤ |*s*|, the notation *s*_[*i*, *j*] _designates the substring *s*_*i *_*s*_*i*+1 _... *s*_*j*_. We note s^
 MathType@MTEF@5@5@+=feaafiart1ev1aaatCvAUfKttLearuWrP9MDH5MBPbIqV92AaeXatLxBI9gBaebbnrfifHhDYfgasaacH8akY=wiFfYdH8Gipec8Eeeu0xXdbba9frFj0=OqFfea0dXdd9vqai=hGuQ8kuc9pgc9s8qqaq=dirpe0xb9q8qiLsFr0=vr0=vr0dc8meaabaqaciaacaGaaeqabaqabeGadaaakeaacuWGZbWCgaqcaaaa@2E2B@ the reverse sequence of *s*, *i.e*.

s^
 MathType@MTEF@5@5@+=feaafiart1ev1aaatCvAUfKttLearuWrP9MDH5MBPbIqV92AaeXatLxBI9gBaebbnrfifHhDYfgasaacH8akY=wiFfYdH8Gipec8Eeeu0xXdbba9frFj0=OqFfea0dXdd9vqai=hGuQ8kuc9pgc9s8qqaq=dirpe0xb9q8qiLsFr0=vr0=vr0dc8meaabaqaciaacaGaaeqabaqabeGadaaakeaacuWGZbWCgaqcaaaa@2E2B@ = *s*_|*s*| _*s*_|*s*|_-1 ...*s*_1_. The set of all sequences of length *l *over A
 MathType@MTEF@5@5@+=feaafiart1ev1aaatCvAUfKttLearuWrP9MDH5MBPbIqV92AaeXatLxBI9gBaebbnrfifHhDYfgasaacH8akY=wiFfYdH8Gipec8Eeeu0xXdbba9frFj0=OqFfea0dXdd9vqai=hGuQ8kuc9pgc9s8qqaq=dirpe0xb9q8qiLsFr0=vr0=vr0dc8meaabaqaciaacaGaaeqabaqabeGadaaakeaat0uy0HwzTfgDPnwy1egaryqtHrhAL1wy0L2yHvdaiqaacqWFaeFqaaa@3820@ is noted Al
 MathType@MTEF@5@5@+=feaafiart1ev1aaatCvAUfKttLearuWrP9MDH5MBPbIqV92AaeXatLxBI9gBaebbnrfifHhDYfgasaacH8akY=wiFfYdH8Gipec8Eeeu0xXdbba9frFj0=OqFfea0dXdd9vqai=hGuQ8kuc9pgc9s8qqaq=dirpe0xb9q8qiLsFr0=vr0=vr0dc8meaabaqaciaacaGaaeqabaqabeGadaaakeaat0uy0HwzTfgDPnwy1egaryqtHrhAL1wy0L2yHvdaiqaacqWFaeFqdaahaaWcbeqaaiabdYgaSbaaaaa@39AE@. Let *s *and *t *be two sequences. A pair of intervals of positions ([*a*, *b*], [*c*, *d*]) is a *diagonal *of (*s*, *t*) if 1 ≤ *a* ≤ *b *≤ |*s*|, 1 ≤ *c *≤ *d *≤ |*t*| and *b *- *a *= *d *- *c*. The first (*resp*. the second) interval of a diagonal *D *of (*s*, *t*) will be designated as the *s-interval *(*resp*. the *t-interval*) of *D*. In order to avoid to deal specifically with some "pathological cases", we allow diagonals to be empty (of length 0).

**Definition 1 ***Let s and t be two sequences. A N-map of s over t is a N-tuple of diagonals of *(*s*, *t*): [([*a*_1_, *b*_1_], [*c*_1_, *d*_1_]), ([*a*_2_, *b*_2_], [*c*_2_, *d*_2_]), ...,([*a*_*N*_, *b*_*N*_], [*c*_*N*_, *d*_*N*_])] *such that *[*a*_*i*_, *b*_*i*_] ∩ [*a*_*j*_, *b*_*j*_] = ∅ *for all *1 ≤ *i, j *≤ *N with j *≠ *i*.

Without loss of generality, we assume in the following that the diagonals of a *N*-map of *s *over *t *are indexed according to the positions of their *s*-intervals. In particular, the *first diagonal *(*resp*. the *last diagonal*) is the one with the smallest (*resp*. the greatest) start position of *s*-interval. Notation Ω(s,t)N
 MathType@MTEF@5@5@+=feaafiart1ev1aaatCvAUfKttLearuWrP9MDH5MBPbIqV92AaeXatLxBI9gBaebbnrfifHhDYfgasaacH8akY=wiFfYdH8Gipec8Eeeu0xXdbba9frFj0=OqFfea0dXdd9vqai=hGuQ8kuc9pgc9s8qqaq=dirpe0xb9q8qiLsFr0=vr0=vr0dc8meaabaqaciaacaGaaeqabaqabeGadaaakeaacqqHPoWvdaqhaaWcbaGaeiikaGIaem4CamNaeiilaWIaemiDaqNaeiykaKcabaGaemOta4eaaaaa@34FE@ denotes the set of all the *N*-maps of *s *over *t*.

**Figure 1:**
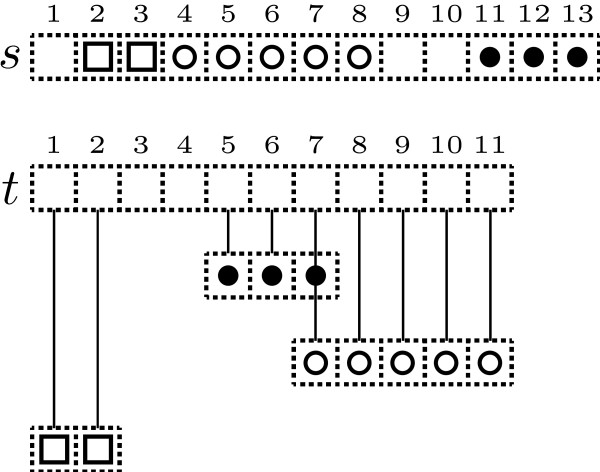
Representation of the 3-map [([2, 3], [1,2]), ([4,8], [7,11]), ([11,13], [5,7])] of *s *over *t*. The positions associated in a diagonal are connected by a line.

**Figure 2:**
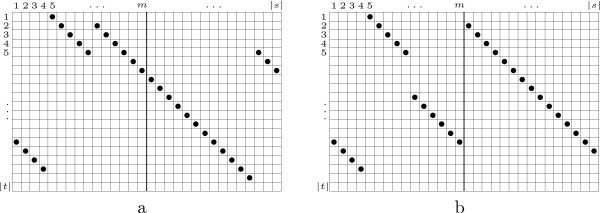
Dotplot representations of two 4-maps. a) Position *m *is inside a diagonal. b) Position *m *is not inside a diagonal.

A *N*-map of *s *over *t *is nothing but a peculiar type of map from a subset of positions of *s *to the set of positions of *t*. In other words, it associates at most one position of *t *to a position of *s*; and none, one or several positions of *s *to a position of *t *(See Figure 1 or Figure 2 for dotplot representation).

A given classical alignment (which is also a map between positions) can be seen, for a certain positive integer *N*, as a *N*-map, both of *s *over *t *and of *t *over *s*. More precisely, an alignment with a fixed number *K *of gaps, like studied in [7-9], is a (*K *+ 1)-map which, with notations of Definition 1, verifies the additional conditions: [*c*_*i*_, *d*_*i*_] ∩ [*c*_*j*_, *d*_*j*_] = ∅ and (*a*_*i *_- *a*_*j*_) × (*c*_*i *_- *c*_*j*_) > 0 for all 1 ≤ *i*, *j *≤ *K *+ 1 with *j *≠ *i*. For 0 <*N *≤ *K *≤ |*s*| and a given *N*-map of *s *over *t*, there is at least one *K*-map defining the same map from positions of *s *to positions of *t*.

Let S
 MathType@MTEF@5@5@+=feaafiart1ev1aaatCvAUfKttLearuWrP9MDH5MBPbIqV92AaeXatLxBI9gBaebbnrfifHhDYfgasaacH8akY=wiFfYdH8Gipec8Eeeu0xXdbba9frFj0=OqFfea0dXdd9vqai=hGuQ8kuc9pgc9s8qqaq=dirpe0xb9q8qiLsFr0=vr0=vr0dc8meaabaqaciaacaGaaeqabaqabeGadaaakeaat0uy0HwzTfgDPnwy1egaryqtHrhAL1wy0L2yHvdaiqaacqWFse=uaaa@3844@ be a *scoring scheme, i.e*. a map from ∪l∈ℕAl×Al
 MathType@MTEF@5@5@+=feaafiart1ev1aaatCvAUfKttLearuWrP9MDH5MBPbIqV92AaeXatLxBI9gBaebbnrfifHhDYfgasaacH8akY=wiFfYdH8Gipec8Eeeu0xXdbba9frFj0=OqFfea0dXdd9vqai=hGuQ8kuc9pgc9s8qqaq=dirpe0xb9q8qiLsFr0=vr0=vr0dc8meaabaqaciaacaGaaeqabaqabeGadaaakeaadaWeqaqaamrtHrhAL1wy0L2yHvtyaeHbnfgDOvwBHrxAJfwnaGabaiab=bq8bnaaCaaaleqabaGaemiBaWgaaOGaey41aqRae8haXh0aaWbaaSqabeaacqWGSbaBaaaabaGaemiBaWMaeyicI48efv3ySLgznfgDOjdarCqr1ngBPrginfgDObcv39gaiuaacqGFveItaeqaniablQIivbaaaa@4E0E@ to ℝ. The score associated to a *N*-map following S
 MathType@MTEF@5@5@+=feaafiart1ev1aaatCvAUfKttLearuWrP9MDH5MBPbIqV92AaeXatLxBI9gBaebbnrfifHhDYfgasaacH8akY=wiFfYdH8Gipec8Eeeu0xXdbba9frFj0=OqFfea0dXdd9vqai=hGuQ8kuc9pgc9s8qqaq=dirpe0xb9q8qiLsFr0=vr0=vr0dc8meaabaqaciaacaGaaeqabaqabeGadaaakeaat0uy0HwzTfgDPnwy1egaryqtHrhAL1wy0L2yHvdaiqaacqWFse=uaaa@3844@ is:

S[([a1,b1],[c1,d1]),...,([aN,bN],[cN,dN])]=∑k=1NS(s[ak,bk],t[ck,dk])
 MathType@MTEF@5@5@+=feaafiart1ev1aaatCvAUfKttLearuWrP9MDH5MBPbIqV92AaeXatLxBI9gBaebbnrfifHhDYfgasaacH8akY=wiFfYdH8Gipec8Eeeu0xXdbba9frFj0=OqFfea0dXdd9vqai=hGuQ8kuc9pgc9s8qqaq=dirpe0xb9q8qiLsFr0=vr0=vr0dc8meaabaqaciaacaGaaeqabaqabeGadaaakeaat0uy0HwzTfgDPnwy1egaryqtHrhAL1wy0L2yHvdaiqaacqWFse=ucqGGBbWwcqGGOaakcqGGBbWwcqWGHbqydaWgaaWcbaGaeGymaedabeaakiabcYcaSiabdkgaInaaBaaaleaacqaIXaqmaeqaaOGaeiyxa0LaeiilaWIaei4waSLaem4yam2aaSbaaSqaaiabigdaXaqabaGccqGGSaalcqWGKbazdaWgaaWcbaGaeGymaedabeaakiabc2faDjabcMcaPiabcYcaSiabc6caUiabc6caUiabc6caUiabcYcaSiabcIcaOiabcUfaBjabdggaHnaaBaaaleaacqWGobGtaeqaaOGaeiilaWIaemOyai2aaSbaaSqaaiabd6eaobqabaGccqGGDbqxcqGGSaalcqGGBbWwcqWGJbWydaWgaaWcbaGaemOta4eabeaakiabcYcaSiabdsgaKnaaBaaaleaacqWGobGtaeqaaOGaeiyxa0LaeiykaKIaeiyxa0Laeyypa0ZaaabCaeaacqWFse=ucqGGOaakcqWGZbWCdaWgaaWcbaGaei4waSLaemyyae2aaSbaaWqaaiabdUgaRbqabaWccqGGSaalcqWGIbGydaWgaaadbaGaem4AaSgabeaaliabc2faDbqabaGccqGGSaalcqWG0baDdaWgaaWcbaGaei4waSLaem4yam2aaSbaaWqaaiabdUgaRbqabaWccqGGSaalcqWGKbazdaWgaaadbaGaem4AaSgabeaaliabc2faDbqabaGccqGGPaqkaSqaaiabdUgaRjabg2da9iabigdaXaqaaiabd6eaobqdcqGHris5aaaa@8817@

As in classical alignment methods, we will consider in the following only additive scoring schemes, *i.e*. defined from a A×A
 MathType@MTEF@5@5@+=feaafiart1ev1aaatCvAUfKttLearuWrP9MDH5MBPbIqV92AaeXatLxBI9gBaebbnrfifHhDYfgasaacH8akY=wiFfYdH8Gipec8Eeeu0xXdbba9frFj0=OqFfea0dXdd9vqai=hGuQ8kuc9pgc9s8qqaq=dirpe0xb9q8qiLsFr0=vr0=vr0dc8meaabaqaciaacaGaaeqabaqabeGadaaakeaat0uy0HwzTfgDPnwy1egaryqtHrhAL1wy0L2yHvdaiqaacqWFaeFqcqGHxdaTcqWFaeFqaaa@3BEE@ substitution matrix *π*, for all lengthes *l *and all pairs of sequences (*u, v*) ∈ Al
 MathType@MTEF@5@5@+=feaafiart1ev1aaatCvAUfKttLearuWrP9MDH5MBPbIqV92AaeXatLxBI9gBaebbnrfifHhDYfgasaacH8akY=wiFfYdH8Gipec8Eeeu0xXdbba9frFj0=OqFfea0dXdd9vqai=hGuQ8kuc9pgc9s8qqaq=dirpe0xb9q8qiLsFr0=vr0=vr0dc8meaabaqaciaacaGaaeqabaqabeGadaaakeaat0uy0HwzTfgDPnwy1egaryqtHrhAL1wy0L2yHvdaiqaacqWFaeFqdaahaaWcbeqaaiabdYgaSbaaaaa@39AE@ × Al
 MathType@MTEF@5@5@+=feaafiart1ev1aaatCvAUfKttLearuWrP9MDH5MBPbIqV92AaeXatLxBI9gBaebbnrfifHhDYfgasaacH8akY=wiFfYdH8Gipec8Eeeu0xXdbba9frFj0=OqFfea0dXdd9vqai=hGuQ8kuc9pgc9s8qqaq=dirpe0xb9q8qiLsFr0=vr0=vr0dc8meaabaqaciaacaGaaeqabaqabeGadaaakeaat0uy0HwzTfgDPnwy1egaryqtHrhAL1wy0L2yHvdaiqaacqWFaeFqdaahaaWcbeqaaiabdYgaSbaaaaa@39AE@, by:

S(u,v)=∑j=1lπ[uj,vj]
 MathType@MTEF@5@5@+=feaafiart1ev1aaatCvAUfKttLearuWrP9MDH5MBPbIqV92AaeXatLxBI9gBaebbnrfifHhDYfgasaacH8akY=wiFfYdH8Gipec8Eeeu0xXdbba9frFj0=OqFfea0dXdd9vqai=hGuQ8kuc9pgc9s8qqaq=dirpe0xb9q8qiLsFr0=vr0=vr0dc8meaabaqaciaacaGaaeqabaqabeGadaaakeaat0uy0HwzTfgDPnwy1egaryqtHrhAL1wy0L2yHvdaiqaacqWFse=ucqGGOaakcqWG1bqDcqGGSaalcqWG2bGDcqGGPaqkcqGH9aqpdaaeWbqaaGGaciab+b8aWnaaBaaaleaacqGGBbWwcqWG1bqDdaWgaaadbaGaemOAaOgabeaaliabcYcaSiabdAha2naaBaaameaacqWGQbGAaeqaaSGaeiyxa0fabeaaaeaacqWGQbGAcqGH9aqpcqaIXaqmaeaacqWGSbaBa0GaeyyeIuoaaaa@5110@

The score of an empty diagonal (*l *= 0) is 0.

The maximum of the scores over all the *N*-maps of *s *over *t *is noted ℳN
 MathType@MTEF@5@5@+=feaafiart1ev1aaatCvAUfKttLearuWrP9MDH5MBPbIqV92AaeXatLxBI9gBaebbnrfifHhDYfgasaacH8akY=wiFfYdH8Gipec8Eeeu0xXdbba9frFj0=OqFfea0dXdd9vqai=hGuQ8kuc9pgc9s8qqaq=dirpe0xb9q8qiLsFr0=vr0=vr0dc8meaabaqaciaacaGaaeqabaqabeGadaaakeaat0uy0HwzTfgDPnwy1egaryqtHrhAL1wy0L2yHvdaiqaacqWFZestdaahaaWcbeqaaiabd6eaobaaaaa@38E1@ (*s*, *t*):

ℳN(s,t)=max⁡Γ∈Ω(s,t)NS(Γ)
 MathType@MTEF@5@5@+=feaafiart1ev1aaatCvAUfKttLearuWrP9MDH5MBPbIqV92AaeXatLxBI9gBaebbnrfifHhDYfgasaacH8akY=wiFfYdH8Gipec8Eeeu0xXdbba9frFj0=OqFfea0dXdd9vqai=hGuQ8kuc9pgc9s8qqaq=dirpe0xb9q8qiLsFr0=vr0=vr0dc8meaabaqaciaacaGaaeqabaqabeGadaaakeaat0uy0HwzTfgDPnwy1egaryqtHrhAL1wy0L2yHvdaiqaacqWFZestdaahaaWcbeqaaiabd6eaobaakiabcIcaOiabdohaZjabcYcaSiabdsha0jabcMcaPiabg2da9maaxababaGagiyBa0MaeiyyaeMaeiiEaGhaleaacqqHtoWrcqGHiiIZcqqHPoWvdaqhaaadbaGaeiikaGIaem4CamNaeiilaWIaemiDaqNaeiykaKcabaGaemOta4eaaaWcbeaakiab=jr8tjabcIcaOiabfo5ahjabcMcaPaaa@540B@

An *optimal N*-map of *s *over *t *is a *N*-map with score ℳN
 MathType@MTEF@5@5@+=feaafiart1ev1aaatCvAUfKttLearuWrP9MDH5MBPbIqV92AaeXatLxBI9gBaebbnrfifHhDYfgasaacH8akY=wiFfYdH8Gipec8Eeeu0xXdbba9frFj0=OqFfea0dXdd9vqai=hGuQ8kuc9pgc9s8qqaq=dirpe0xb9q8qiLsFr0=vr0=vr0dc8meaabaqaciaacaGaaeqabaqabeGadaaakeaat0uy0HwzTfgDPnwy1egaryqtHrhAL1wy0L2yHvdaiqaacqWFZestdaahaaWcbeqaaiabd6eaobaaaaa@38E1@ (*s*, *t*). By convention, a 0-map is the empty set and ℳ0
 MathType@MTEF@5@5@+=feaafiart1ev1aaatCvAUfKttLearuWrP9MDH5MBPbIqV92AaeXatLxBI9gBaebbnrfifHhDYfgasaacH8akY=wiFfYdH8Gipec8Eeeu0xXdbba9frFj0=OqFfea0dXdd9vqai=hGuQ8kuc9pgc9s8qqaq=dirpe0xb9q8qiLsFr0=vr0=vr0dc8meaabaqaciaacaGaaeqabaqabeGadaaakeaat0uy0HwzTfgDPnwy1egaryqtHrhAL1wy0L2yHvdaiqaacqWFZestdaahaaWcbeqaaiabicdaWaaaaaa@38AA@ (*s*, *t*) = 0.

Depending on whether the substitution matrix contains negative values or not, the optimal *N*-map is said to be local or global. These concepts are used by analogy with the case of alignment. When the matrix contains only non-negative values (global case), a corresponding optimal *N*-map of *s *over *t *will attempt to associate each position of *s *with a position of *t*, as in a global alignment. When the matrix contains some negative values (local case), the optimal *N*-map will be reached by considering only subparts of *s *which lead to a positive contribution of the total score when associated with a segment of *t*, once more as in local alignment. Basically, a global *N*-map of *s *over *t *spans the entire length of *s *(except possibly some boundary positions) while a local *N*-map identifies *N *non-overlapping segments of *s *with maximum scores against *t*.

Some pathological situations could arise in the local case. In particular there could be some positions *i *of *s *such that *π *[*s*_*i*_, *t*_*j*_] is negative for all positions *j *of *t*. Without considering empty diagonals, ℳN
 MathType@MTEF@5@5@+=feaafiart1ev1aaatCvAUfKttLearuWrP9MDH5MBPbIqV92AaeXatLxBI9gBaebbnrfifHhDYfgasaacH8akY=wiFfYdH8Gipec8Eeeu0xXdbba9frFj0=OqFfea0dXdd9vqai=hGuQ8kuc9pgc9s8qqaq=dirpe0xb9q8qiLsFr0=vr0=vr0dc8meaabaqaciaacaGaaeqabaqabeGadaaakeaat0uy0HwzTfgDPnwy1egaryqtHrhAL1wy0L2yHvdaiqaacqWFZestdaahaaWcbeqaaiabd6eaobaaaaa@38E1@ (*s*, *t*) would be not always growing with *N*.

The current software implementation incorporates various substitution matrices in particular for amino acids (PAM, BLOSUM, *etc*.).

For handling inversions, which is not allowed by Definition 1, it is first needed to extend this definition by adding a sign to the pair of intervals:

• (+, [*a*, *b*], [*c*, *d*]) means that the positions [(*a*, *c*), (*a *+ 1, *c *+ 1),...] are associated (normal case),

• (-, [*a*, *b*], [*c*, *d*]) means that the positions [(*a*, *d*), (*a *+ 1, *d *- 1),...] are associated (inversion case).

Another required extension concerns the way of calculating the score for "reverse diagonals". This point depends on the nature of sequences. For instance in the case of DNA sequences, the score of (-, [*a*, *b*], [*c*, *d*]) is computed by summing the individual substitution scores of *s*_[*a*, *b*] _against the complementary-reverse of *t*_[*c*, *d*]_. If *s *and *t *are sequences of genes, this score is obtained by considering *s*_[*a*, *b*] _against the reverse of *t*_[*c*, *d*]_.

## 2 Algorithms

Given two sequences *s*, *t *and a positive integer *N*, we address two problems:

• **Problem 1**: computing the optimal scores ℳK
 MathType@MTEF@5@5@+=feaafiart1ev1aaatCvAUfKttLearuWrP9MDH5MBPbIqV92AaeXatLxBI9gBaebbnrfifHhDYfgasaacH8akY=wiFfYdH8Gipec8Eeeu0xXdbba9frFj0=OqFfea0dXdd9vqai=hGuQ8kuc9pgc9s8qqaq=dirpe0xb9q8qiLsFr0=vr0=vr0dc8meaabaqaciaacaGaaeqabaqabeGadaaakeaat0uy0HwzTfgDPnwy1egaryqtHrhAL1wy0L2yHvdaiqaacqWFZestdaahaaWcbeqaaiabdUealbaaaaa@38DB@ (*s*, *t*) with *K *running from 1 to *N*.

• **Problem 2**: outputting the diagonals of an optimal *N*-map.

### Computing the optimal scores

Let Best_[*i*, *j*, *K*] _be the maximal score obtained by a *K*-map of *s*_[1, *i*] _over *t ending at *(*i*, *j*), *i.e*. such that its last diagonal ([*a*_*K*_, *b*_*K*_], [*c*_*K*_, *d*_*K*_]) verifies *b*_*K *_= *i *and *d*_*K *_= *j*. By setting Best_[0, *j*, *K*]_, Best_[*i*, 0 , *K*] _and Best_[*i*, *j*, 0] _to 0 for all integers *i*, *j *and *K*, we have the following recurrence relation:

Best[i+1,j+1,K]=π[si+1,tj+1]+max⁡{Best[i,j,K],max⁡k≤i,l≤|t|Best[k,l,K−1]}
 MathType@MTEF@5@5@+=feaafiart1ev1aaatCvAUfKttLearuWrP9MDH5MBPbIqV92AaeXatLxBI9gBaebbnrfifHhDYfgasaacH8akY=wiFfYdH8Gipec8Eeeu0xXdbba9frFj0=OqFfea0dXdd9vqai=hGuQ8kuc9pgc9s8qqaq=dirpe0xb9q8qiLsFr0=vr0=vr0dc8meaabaqaciaacaGaaeqabaqabeGadaaakeaacqqGcbGqcqqGLbqzcqqGZbWCcqqG0baDdaWgaaWcbaGaei4waSLaemyAaKMaey4kaSIaeGymaeJaeiilaWIaemOAaOMaey4kaSIaeGymaeJaeiilaWIaem4saSKaeiyxa0fabeaakiabg2da9GGaciab=b8aWnaaBaaaleaacqGGBbWwcqWGZbWCdaWgaaadbaGaemyAaKMaey4kaSIaeGymaedabeaaliabcYcaSiabdsha0naaBaaameaacqWGQbGAcqGHRaWkcqaIXaqmaeqaaSGaeiyxa0fabeaakiabgUcaRiGbc2gaTjabcggaHjabcIha4naacmaabaGaeeOqaiKaeeyzauMaee4CamNaeeiDaqNaaCjaVpaaBaaaleaacqGGBbWwcqWGPbqAcqGGSaalcqWGQbGAcqGGSaalcqWGlbWscqGGDbqxaeqaaOGaeiilaWYaaCbeaeaacyGGTbqBcqGGHbqycqGG4baEaSqaaiabdUgaRjabgsMiJkabdMgaPjabcYcaSiabdYgaSjabgsMiJoaaemaabaGaemiDaqhacaGLhWUaayjcSdaabeaakiabbkeacjabbwgaLjabbohaZjabbsha0naaBaaaleaacqGGBbWwcqWGRbWAcqGGSaalcqWGSbaBcqGGSaalcqWGlbWscqGHsislcqaIXaqmcqGGDbqxaeqaaaGccaGL7bGaayzFaaGaaCjaVdaa@875A@

The correctness of this relation is straightforwardly proved by induction. Let us consider the maximum involved in the right part of this equation. It is equal to:

• Best_[*i*, *j*, *K*]_, if the greatest score is obtained by incrementing the length of the last diagonal of an optimal *K*-map of *s*_[1, *i*] _over *t *ending at (*i*, *j*) – then the last diagonal will end at (*i *+ 1, *j *+ 1).

• max⁡k≤i,l≤jBest[k,l,K−1]
 MathType@MTEF@5@5@+=feaafiart1ev1aaatCvAUfKttLearuWrP9MDH5MBPbIqV92AaeXatLxBI9gBaebbnrfifHhDYfgasaacH8akY=wiFfYdH8Gipec8Eeeu0xXdbba9frFj0=OqFfea0dXdd9vqai=hGuQ8kuc9pgc9s8qqaq=dirpe0xb9q8qiLsFr0=vr0=vr0dc8meaabaqaciaacaGaaeqabaqabeGadaaakeaadaWfqaqaaiGbc2gaTjabcggaHjabcIha4bWcbaGaem4AaSMaeyizImQaemyAaKMaeiilaWIaemiBaWMaeyizImQaemOAaOgabeaakiabbkeacjabbwgaLjabbohaZjabbsha0naaBaaaleaacqGGBbWwcqWGRbWAcqGGSaalcqWGSbaBcqGGSaalcqWGlbWscqGHsislcqaIXaqmcqGGDbqxaeqaaaaa@4A37@, if the greatest score is obtained by adding the diagonal of length 1 ([*i *+ 1, *i *+ 1], [*j *+ 1, *j *+ 1]) to an optimal (*K *- 1)-map of *s*_[1, *i*] _over *t*.

To compute the entries of Best referred to the index (*i *+ 1), we only need to know the entries referred to the index *i*. Thus computing the optimal scores of all the *K*-maps of *s *over *t*, with *K *from 1 to *N*, can be done in *O *(|*s*| × |*t*| × *N*) time using *O *(|*t*| × *N*) memory space to store the dynamic programming variables. We can introduce now the formal algorithm **Alg_1 **which solves the problem of computing the optimal scores for global or local *N*-maps without inversion.

Algorithm **Alg_1 **takes as input two sequences *s*, *t *and a number of parts *N *and returns:

• B_s _and B_l_, two |*t*| × *N *matrices where the entry B_s[*j*, *K*] _contains the maximal score of a *K*-map of *s *over *t *ending at (|*s*|, *j*) – with the preceding notations B_s[*j*, *K*] _= Best_[|*s*|, *j*, *K*] _– and the entry B_l[*j*, *K*] _stores the length of the last diagonal of a *K*-map ending at (|*s*|, *j*) with score B_s[*j*, *K*]_;

• M_s _and M_d_, two arrays of size *N *where the entry M_s[*K*] _stores the optimal score of a *K*-map of *s *over *t *– with the preceding notations M_s[*K*] _= ℳK
 MathType@MTEF@5@5@+=feaafiart1ev1aaatCvAUfKttLearuWrP9MDH5MBPbIqV92AaeXatLxBI9gBaebbnrfifHhDYfgasaacH8akY=wiFfYdH8Gipec8Eeeu0xXdbba9frFj0=OqFfea0dXdd9vqai=hGuQ8kuc9pgc9s8qqaq=dirpe0xb9q8qiLsFr0=vr0=vr0dc8meaabaqaciaacaGaaeqabaqabeGadaaakeaat0uy0HwzTfgDPnwy1egaryqtHrhAL1wy0L2yHvdaiqaacqWFZestdaahaaWcbeqaaiabdUealbaaaaa@38DB@ (*s*, *t*) = max_*q *≤ |*s*|, *p *≤ |*t*| _Best_[*q*, *p*, *K*] _– and the entry M_d[*K*] _stores the last diagonal of a *K*-map with score M_s[*K*]_.

The correctness of Algorithm **Alg_1 **is proved by induction over the positions of *s*. The time and memory space complexities are straightforwardly analyzed.

The variables B_l _and M_d _are not involved in the computation of the maximal scores of *K*-maps for 1 ≤ *K *≤ *N *(they will be used by the algorithm in charge of outputting the diagonals). If we are only interested in solving Problem 1, these variables as well as the lines 7, 10, 14 and 19 can be deleted. Algorithm **Alg_1 **will still return the optimal scores of *K*-maps with 0 ≤ *K *≤ *N *in the array M_s_.

**Algorithm 1 Alg_1 **(*s*, *t*, *N*, B_s_, B_l_, M_s_, M_d_)

1: B_s[*j*, *K*] _← 0 ; B_l[*j*, *K*] _← 0 ; M_s[*K*] _← 0 ; M_d[*K*] _← NULL ; (*j *= 0 ... |*t*|, *K *= 0 ... *N*)

2: **for ***i *= 1 **to **|*s*| **do**

3:   **for ***j *= 1 **to **|*t*| **do**

4:      **for ***K *= *N ***to **1 **do**

5:         **if **B_s[*j*-1, *K*] _≥ M_s[*k*-1] _**then**

6:            B′s[j,K]
 MathType@MTEF@5@5@+=feaafiart1ev1aaatCvAUfKttLearuWrP9MDH5MBPbIqV92AaeXatLxBI9gBaebbnrfifHhDYfgasaacH8akY=wiFfYdH8Gipec8Eeeu0xXdbba9frFj0=OqFfea0dXdd9vqai=hGuQ8kuc9pgc9s8qqaq=dirpe0xb9q8qiLsFr0=vr0=vr0dc8meaabaqaciaacaGaaeqabaqabeGadaaakeaacuqGcbGqgaqbamaaBaaaleaacqqGZbWCcqGGBbWwcqWGQbGAcqGGSaalcqWGlbWscqGGDbqxaeqaaaaa@3538@ ← *π*_[*s*_*i*_, *t*_*j*_] _+ B_s[*j*-1, *K*] _;

7:            B′l[j,K]
 MathType@MTEF@5@5@+=feaafiart1ev1aaatCvAUfKttLearuWrP9MDH5MBPbIqV92AaeXatLxBI9gBaebbnrfifHhDYfgasaacH8akY=wiFfYdH8Gipec8Eeeu0xXdbba9frFj0=OqFfea0dXdd9vqai=hGuQ8kuc9pgc9s8qqaq=dirpe0xb9q8qiLsFr0=vr0=vr0dc8meaabaqaciaacaGaaeqabaqabeGadaaakeaacuqGcbGqgaqbamaaBaaaleaacqqGSbaBcqGGBbWwcqWGQbGAcqGGSaalcqWGlbWscqGGDbqxaeqaaaaa@352A@ ← B_l[*j*-1, *K*] _+ 1 ;

8:         **else**

9:            B′s[j,K]
 MathType@MTEF@5@5@+=feaafiart1ev1aaatCvAUfKttLearuWrP9MDH5MBPbIqV92AaeXatLxBI9gBaebbnrfifHhDYfgasaacH8akY=wiFfYdH8Gipec8Eeeu0xXdbba9frFj0=OqFfea0dXdd9vqai=hGuQ8kuc9pgc9s8qqaq=dirpe0xb9q8qiLsFr0=vr0=vr0dc8meaabaqaciaacaGaaeqabaqabeGadaaakeaacuqGcbGqgaqbamaaBaaaleaacqqGZbWCcqGGBbWwcqWGQbGAcqGGSaalcqWGlbWscqGGDbqxaeqaaaaa@3538@ ← *π*_[*s*_*i*_, *t*_*j*_] _+ M_s[*K*-1] _;

10:            B′l[j,K]
 MathType@MTEF@5@5@+=feaafiart1ev1aaatCvAUfKttLearuWrP9MDH5MBPbIqV92AaeXatLxBI9gBaebbnrfifHhDYfgasaacH8akY=wiFfYdH8Gipec8Eeeu0xXdbba9frFj0=OqFfea0dXdd9vqai=hGuQ8kuc9pgc9s8qqaq=dirpe0xb9q8qiLsFr0=vr0=vr0dc8meaabaqaciaacaGaaeqabaqabeGadaaakeaacuqGcbGqgaqbamaaBaaaleaacqqGSbaBcqGGBbWwcqWGQbGAcqGGSaalcqWGlbWscqGGDbqxaeqaaaaa@352A@ ← 1;

11:         **end if**

12:         **if **B′s[j,K]
 MathType@MTEF@5@5@+=feaafiart1ev1aaatCvAUfKttLearuWrP9MDH5MBPbIqV92AaeXatLxBI9gBaebbnrfifHhDYfgasaacH8akY=wiFfYdH8Gipec8Eeeu0xXdbba9frFj0=OqFfea0dXdd9vqai=hGuQ8kuc9pgc9s8qqaq=dirpe0xb9q8qiLsFr0=vr0=vr0dc8meaabaqaciaacaGaaeqabaqabeGadaaakeaacuqGcbGqgaqbamaaBaaaleaacqqGZbWCcqGGBbWwcqWGQbGAcqGGSaalcqWGlbWscqGGDbqxaeqaaaaa@3538@ ≥ M_s[*K*] _**then**

13:            M_s[*K*] _← B′s[j,K]
 MathType@MTEF@5@5@+=feaafiart1ev1aaatCvAUfKttLearuWrP9MDH5MBPbIqV92AaeXatLxBI9gBaebbnrfifHhDYfgasaacH8akY=wiFfYdH8Gipec8Eeeu0xXdbba9frFj0=OqFfea0dXdd9vqai=hGuQ8kuc9pgc9s8qqaq=dirpe0xb9q8qiLsFr0=vr0=vr0dc8meaabaqaciaacaGaaeqabaqabeGadaaakeaacuqGcbGqgaqbamaaBaaaleaacqqGZbWCcqGGBbWwcqWGQbGAcqGGSaalcqWGlbWscqGGDbqxaeqaaaaa@3538@ ;

14:            M_d[*K*] _← ([*i *- B′l[j,K]
 MathType@MTEF@5@5@+=feaafiart1ev1aaatCvAUfKttLearuWrP9MDH5MBPbIqV92AaeXatLxBI9gBaebbnrfifHhDYfgasaacH8akY=wiFfYdH8Gipec8Eeeu0xXdbba9frFj0=OqFfea0dXdd9vqai=hGuQ8kuc9pgc9s8qqaq=dirpe0xb9q8qiLsFr0=vr0=vr0dc8meaabaqaciaacaGaaeqabaqabeGadaaakeaacuqGcbGqgaqbamaaBaaaleaacqqGSbaBcqGGBbWwcqWGQbGAcqGGSaalcqWGlbWscqGGDbqxaeqaaaaa@352A@ + 1, *i*], [*j *- B′l[j,K]
 MathType@MTEF@5@5@+=feaafiart1ev1aaatCvAUfKttLearuWrP9MDH5MBPbIqV92AaeXatLxBI9gBaebbnrfifHhDYfgasaacH8akY=wiFfYdH8Gipec8Eeeu0xXdbba9frFj0=OqFfea0dXdd9vqai=hGuQ8kuc9pgc9s8qqaq=dirpe0xb9q8qiLsFr0=vr0=vr0dc8meaabaqaciaacaGaaeqabaqabeGadaaakeaacuqGcbGqgaqbamaaBaaaleaacqqGSbaBcqGGBbWwcqWGQbGAcqGGSaalcqWGlbWscqGGDbqxaeqaaaaa@352A@ + 1, *j*]) ;

15:         **end if**

16:      **end for**

17:   **end for**

18:   **swap **(B_s_, B′s
 MathType@MTEF@5@5@+=feaafiart1ev1aaatCvAUfKttLearuWrP9MDH5MBPbIqV92AaeXatLxBI9gBaebbnrfifHhDYfgasaacH8akY=wiFfYdH8Gipec8Eeeu0xXdbba9frFj0=OqFfea0dXdd9vqai=hGuQ8kuc9pgc9s8qqaq=dirpe0xb9q8qiLsFr0=vr0=vr0dc8meaabaqaciaacaGaaeqabaqabeGadaaakeaacuqGcbGqgaqbamaaBaaaleaacqqGZbWCaeqaaaaa@2F5C@) ;

19:   **swap **(B_l_, B′l
 MathType@MTEF@5@5@+=feaafiart1ev1aaatCvAUfKttLearuWrP9MDH5MBPbIqV92AaeXatLxBI9gBaebbnrfifHhDYfgasaacH8akY=wiFfYdH8Gipec8Eeeu0xXdbba9frFj0=OqFfea0dXdd9vqai=hGuQ8kuc9pgc9s8qqaq=dirpe0xb9q8qiLsFr0=vr0=vr0dc8meaabaqaciaacaGaaeqabaqabeGadaaakeaacuqGcbGqgaqbamaaBaaaleaacqqGSbaBaeqaaaaa@2F4E@) ;

20: **end for**

**Theorem 1 ***Algorithm ***Alg_1 ***computes the optimal score of the K-maps of a sequence s over a sequence t, for K from *1 *to N, in time O *(|*s*| × |*t*| × *N*) *using O *(|*s*| + |*t*| × *N*) *memory space*.

### Outputting the diagonals of an optimal *N*-map

Before presenting the formal algorithm, we need to introduce some additional notations and results about "dividing maps".

We say that a position *m *of *s *is *inside a diagonal *of a *N*-map Γ if there is a diagonal ([*a*, *b*], [*c*, *d*]) ∈ Γ such that *a *≤ *m *<*b *(Figure 2a). This notion excludes two cases:

1. when *m *is not contained by any diagonal (this is usual with local *N*-maps),

2. when a diagonal is exactly ending at *m *in its first interval.

We denote as Best¯[i,j,K]
 MathType@MTEF@5@5@+=feaafiart1ev1aaatCvAUfKttLearuWrP9MDH5MBPbIqV92AaeXatLxBI9gBaebbnrfifHhDYfgasaacH8akY=wiFfYdH8Gipec8Eeeu0xXdbba9frFj0=OqFfea0dXdd9vqai=hGuQ8kuc9pgc9s8qqaq=dirpe0xb9q8qiLsFr0=vr0=vr0dc8meaabaqaciaacaGaaeqabaqabeGadaaakeaadaqdaaqaaiabbkeacjabbwgaLjabbohaZjabbsha0baadaWgaaWcbaGaei4waSLaemyAaKMaeiilaWIaemOAaOMaeiilaWIaem4saSKaeiyxa0fabeaaaaa@3A38@ the maximal score obtained by a *K*-map of *s*_[*i*,|*s*|] _over *t*_[*j*,|*t*|] _*starting at *(*i*, *j*), *i.e*. such that its first diagonal ([*a*_1_, *b*_1_], [*c*_1_, *d*_1_]) verifies *a*_1 _= *i *and *c*_1 _= *j*.

**Lemma 1 ***Let s and t be two sequences, m a position of s and N a positive integer. The optimal score of a N-map *ℳN
 MathType@MTEF@5@5@+=feaafiart1ev1aaatCvAUfKttLearuWrP9MDH5MBPbIqV92AaeXatLxBI9gBaebbnrfifHhDYfgasaacH8akY=wiFfYdH8Gipec8Eeeu0xXdbba9frFj0=OqFfea0dXdd9vqai=hGuQ8kuc9pgc9s8qqaq=dirpe0xb9q8qiLsFr0=vr0=vr0dc8meaabaqaciaacaGaaeqabaqabeGadaaakeaat0uy0HwzTfgDPnwy1egaryqtHrhAL1wy0L2yHvdaiqaacqWFZestdaahaaWcbeqaaiabd6eaobaaaaa@38E1@ (*s*, *t*) *is equal to the maximum of the two following quantities:*

• Q1=max⁡1≤K≤N;1≤p<|t|{Best[m,p,K]+Best¯[m+1,p+1,N−K+1]}
 MathType@MTEF@5@5@+=feaafiart1ev1aaatCvAUfKttLearuWrP9MDH5MBPbIqV92AaeXatLxBI9gBaebbnrfifHhDYfgasaacH8akY=wiFfYdH8Gipec8Eeeu0xXdbba9frFj0=OqFfea0dXdd9vqai=hGuQ8kuc9pgc9s8qqaq=dirpe0xb9q8qiLsFr0=vr0=vr0dc8meaabaqaciaacaGaaeqabaqabeGadaaakeaat0uy0HwzTfgDPnwy1egaryqtHrhAL1wy0L2yHvdaiqaacqWFqeFudaWgaaWcbaGaeGymaedabeaakiabg2da9maaxababaGagiyBa0MaeiyyaeMaeiiEaGhaleaacqaIXaqmcqGHKjYOcqWGlbWscqGHKjYOcqWGobGtcqGG7aWocqaIXaqmcqGHKjYOcqWGWbaCcqGH8aapdaabdaqaaiabdsha0bGaay5bSlaawIa7aaqabaGccqGG7bWEcqqGcbGqcqqGLbqzcqqGZbWCcqqG0baDdaWgaaWcbaGaei4waSLaemyBa0MaeiilaWIaemiCaaNaeiilaWIaem4saSKaeiyxa0fabeaakiabgUcaRmaanaaabaGaeeOqaiKaeeyzauMaee4CamNaeeiDaqhaamaaBaaaleaacqGGBbWwcqWGTbqBcqGHRaWkcqaIXaqmcqGGSaalcqWGWbaCcqGHRaWkcqaIXaqmcqGGSaalcqWGobGtcqGHsislcqWGlbWscqGHRaWkcqaIXaqmcqGGDbqxaeqaaOGaeiyFa0haaa@76C3@

• Q2=max⁡1≤K≤N{ℳK(s[1,m],t)+ℳN−K(s[m+1,|s|],t)}
 MathType@MTEF@5@5@+=feaafiart1ev1aaatCvAUfKttLearuWrP9MDH5MBPbIqV92AaeXatLxBI9gBaebbnrfifHhDYfgasaacH8akY=wiFfYdH8Gipec8Eeeu0xXdbba9frFj0=OqFfea0dXdd9vqai=hGuQ8kuc9pgc9s8qqaq=dirpe0xb9q8qiLsFr0=vr0=vr0dc8meaabaqaciaacaGaaeqabaqabeGadaaakeaat0uy0HwzTfgDPnwy1egaryqtHrhAL1wy0L2yHvdaiqaacqWFqeFudaWgaaWcbaGaeGOmaidabeaakiabg2da9maaxababaGagiyBa0MaeiyyaeMaeiiEaGhaleaacqaIXaqmcqGHKjYOcqWGlbWscqGHKjYOcqWGobGtaeqaaOGaei4EaSNae83mH00aaWbaaSqabeaaieGacqGFlbWsaaGccqGGOaakcqWGZbWCdaWgaaWcbaGaei4waSLaeGymaeJaeiilaWIaemyBa0Maeiyxa0fabeaakiabcYcaSiabdsha0jabcMcaPiabgUcaRiab=ntinnaaCaaaleqabaGaemOta4KaeyOeI0Iaem4saSeaaOGaeiikaGIaem4Cam3aaSbaaSqaaiabcUfaBjabd2gaTjabgUcaRiabigdaXiabcYcaSiabcYha8jabdohaZjabcYha8jabc2faDbqabaGccqGGSaalcqWG0baDcqGGPaqkcqGG9bqFaaa@6C6E@

**Proof: **Let Γ = [([*a*_1_, *b*_1_], [*c*_1_, *d*_1_]), ..., ([*a*_*N*_, *b*_*N*_], [*c*_*N*_, *d*_*N*_])] be a *N*-map of *s *over *t *with score ℳN
 MathType@MTEF@5@5@+=feaafiart1ev1aaatCvAUfKttLearuWrP9MDH5MBPbIqV92AaeXatLxBI9gBaebbnrfifHhDYfgasaacH8akY=wiFfYdH8Gipec8Eeeu0xXdbba9frFj0=OqFfea0dXdd9vqai=hGuQ8kuc9pgc9s8qqaq=dirpe0xb9q8qiLsFr0=vr0=vr0dc8meaabaqaciaacaGaaeqabaqabeGadaaakeaat0uy0HwzTfgDPnwy1egaryqtHrhAL1wy0L2yHvdaiqaacqWFZestdaahaaWcbeqaaiabd6eaobaaaaa@38E1@ (*s*, *t*). There are two possibilities: either the position *m *is inside a diagonal *K *of Γ, or not. In the first case, there are a *K*-map Γ*' *ending at [*m*, *c*_*K *_+ *m *- *a*_*K*_] and a (*N *- *K *+ 1)-map Γ*" *starting at position [*m *+ 1, *c*_*K *_+ *m *- *a*_*K *_+ 1] such that S(Γ′)+S(Γ″)=ℳN(s,t)
 MathType@MTEF@5@5@+=feaafiart1ev1aaatCvAUfKttLearuWrP9MDH5MBPbIqV92AaeXatLxBI9gBaebbnrfifHhDYfgasaacH8akY=wiFfYdH8Gipec8Eeeu0xXdbba9frFj0=OqFfea0dXdd9vqai=hGuQ8kuc9pgc9s8qqaq=dirpe0xb9q8qiLsFr0=vr0=vr0dc8meaabaqaciaacaGaaeqabaqabeGadaaakeaat0uy0HwzTfgDPnwy1egaryqtHrhAL1wy0L2yHvdaiqaacqWFse=ucqGGOaakcuqHtoWrgaqbaiabcMcaPiabgUcaRiab=jr8tjabcIcaOiqbfo5ahzaagaGaeiykaKIaeyypa0Jae83mH00aaWbaaSqabeaacqWGobGtaaGccqGGOaakcqWGZbWCcqGGSaalcqWG0baDcqGGPaqkaaa@4A48@, which implies ℳN(s,t)≤Q1
 MathType@MTEF@5@5@+=feaafiart1ev1aaatCvAUfKttLearuWrP9MDH5MBPbIqV92AaeXatLxBI9gBaebbnrfifHhDYfgasaacH8akY=wiFfYdH8Gipec8Eeeu0xXdbba9frFj0=OqFfea0dXdd9vqai=hGuQ8kuc9pgc9s8qqaq=dirpe0xb9q8qiLsFr0=vr0=vr0dc8meaabaqaciaacaGaaeqabaqabeGadaaakeaat0uy0HwzTfgDPnwy1egaryqtHrhAL1wy0L2yHvdaiqaacqWFZestdaahaaWcbeqaaiabd6eaobaakiabcIcaOiabdohaZjabcYcaSiabdsha0jabcMcaPiabgsMiJkab=br8rnaaBaaaleaacqaIXaqmaeqaaaaa@4305@. In the second case, let *K *be such that *m *= *b*_*K *_or *b*_*K *_<*m *<*a*_*K*+1_. There is a *K*-map Γ*' *of *s*_[1, *m*] _over *t *and a (*N *- *K*)-map Γ*" *of *s*_[*m*+1,|*s*|] _over *t *such that S(Γ′)+S(Γ″)=ℳN(s,t)
 MathType@MTEF@5@5@+=feaafiart1ev1aaatCvAUfKttLearuWrP9MDH5MBPbIqV92AaeXatLxBI9gBaebbnrfifHhDYfgasaacH8akY=wiFfYdH8Gipec8Eeeu0xXdbba9frFj0=OqFfea0dXdd9vqai=hGuQ8kuc9pgc9s8qqaq=dirpe0xb9q8qiLsFr0=vr0=vr0dc8meaabaqaciaacaGaaeqabaqabeGadaaakeaat0uy0HwzTfgDPnwy1egaryqtHrhAL1wy0L2yHvdaiqaacqWFse=ucqGGOaakcuqHtoWrgaqbaiabcMcaPiabgUcaRiab=jr8tjabcIcaOiqbfo5ahzaagaGaeiykaKIaeyypa0Jae83mH00aaWbaaSqabeaacqWGobGtaaGccqGGOaakcqWGZbWCcqGGSaalcqWG0baDcqGGPaqkaaa@4A48@, which implies ℳN(s,t)≤Q2
 MathType@MTEF@5@5@+=feaafiart1ev1aaatCvAUfKttLearuWrP9MDH5MBPbIqV92AaeXatLxBI9gBaebbnrfifHhDYfgasaacH8akY=wiFfYdH8Gipec8Eeeu0xXdbba9frFj0=OqFfea0dXdd9vqai=hGuQ8kuc9pgc9s8qqaq=dirpe0xb9q8qiLsFr0=vr0=vr0dc8meaabaqaciaacaGaaeqabaqabeGadaaakeaat0uy0HwzTfgDPnwy1egaryqtHrhAL1wy0L2yHvdaiqaacqWFZestdaahaaWcbeqaaiabd6eaobaakiabcIcaOiabdohaZjabcYcaSiabdsha0jabcMcaPiabgsMiJkab=br8rnaaBaaaleaacqaIYaGmaeqaaaaa@4307@. In both cases, ℳN
 MathType@MTEF@5@5@+=feaafiart1ev1aaatCvAUfKttLearuWrP9MDH5MBPbIqV92AaeXatLxBI9gBaebbnrfifHhDYfgasaacH8akY=wiFfYdH8Gipec8Eeeu0xXdbba9frFj0=OqFfea0dXdd9vqai=hGuQ8kuc9pgc9s8qqaq=dirpe0xb9q8qiLsFr0=vr0=vr0dc8meaabaqaciaacaGaaeqabaqabeGadaaakeaat0uy0HwzTfgDPnwy1egaryqtHrhAL1wy0L2yHvdaiqaacqWFZestdaahaaWcbeqaaiabd6eaobaaaaa@38E1@ (*s*, *t*) is smaller than max{Q1,Q2
 MathType@MTEF@5@5@+=feaafiart1ev1aaatCvAUfKttLearuWrP9MDH5MBPbIqV92AaeXatLxBI9gBaebbnrfifHhDYfgasaacH8akY=wiFfYdH8Gipec8Eeeu0xXdbba9frFj0=OqFfea0dXdd9vqai=hGuQ8kuc9pgc9s8qqaq=dirpe0xb9q8qiLsFr0=vr0=vr0dc8meaabaqaciaacaGaaeqabaqabeGadaaakeaat0uy0HwzTfgDPnwy1egaryqtHrhAL1wy0L2yHvdaiqaacqWFqeFudaWgaaWcbaGaeGymaedabeaakiabcYcaSiab=br8rnaaBaaaleaacqaIYaGmaeqaaaaa@3D3B@}.

On the other hand, for all integers 1 ≤ *K *≤ *N*, for all positions 1 ≤ *p *< |*t*|, for all *K*-maps Γ′=[([a′1,b′1],[c′1,d′1]),...,([a′K,m],[c′K,p])]
 MathType@MTEF@5@5@+=feaafiart1ev1aaatCvAUfKttLearuWrP9MDH5MBPbIqV92AaeXatLxBI9gBaebbnrfifHhDYfgasaacH8akY=wiFfYdH8Gipec8Eeeu0xXdbba9frFj0=OqFfea0dXdd9vqai=hGuQ8kuc9pgc9s8qqaq=dirpe0xb9q8qiLsFr0=vr0=vr0dc8meaabaqaciaacaGaaeqabaqabeGadaaakeaacuqHtoWrgaqbaiabg2da9iabcUfaBjabcIcaOiabcUfaBjqbdggaHzaafaWaaSbaaSqaaiabigdaXaqabaGccqGGSaalcuWGIbGygaqbamaaBaaaleaacqaIXaqmaeqaaOGaeiyxa0LaeiilaWIaei4waSLafm4yamMbauaadaWgaaWcbaGaeGymaedabeaakiabcYcaSiqbdsgaKzaafaWaaSbaaSqaaiabigdaXaqabaGccqGGDbqxcqGGPaqkcqGGSaalcqGGUaGlcqGGUaGlcqGGUaGlcqGGSaalcqGGOaakcqGGBbWwcuWGHbqygaqbamaaBaaaleaacqWGlbWsaeqaaOGaeiilaWIaemyBa0Maeiyxa0LaeiilaWIaei4waSLafm4yamMbauaadaWgaaWcbaGaem4saSeabeaakiabcYcaSiabdchaWjabc2faDjabcMcaPiabc2faDbaa@5ADE@ and for all (*N *- *K *+ 1)-maps Γ″=[([m+1,b″1],[p+1,d″1]),...,([a″N−K+1,b″N−K+1],[c″N−K+1,d″N−K+1])]
 MathType@MTEF@5@5@+=feaafiart1ev1aaatCvAUfKttLearuWrP9MDH5MBPbIqV92AaeXatLxBI9gBaebbnrfifHhDYfgasaacH8akY=wiFfYdH8Gipec8Eeeu0xXdbba9frFj0=OqFfea0dXdd9vqai=hGuQ8kuc9pgc9s8qqaq=dirpe0xb9q8qiLsFr0=vr0=vr0dc8meaabaqaciaacaGaaeqabaqabeGadaaakeaacuqHtoWrgaGbaiabg2da9iabcUfaBjabcIcaOiabcUfaBjabd2gaTjabgUcaRiabigdaXiabcYcaSiqbdkgaIzaagaWaaSbaaSqaaiabigdaXaqabaGccqGGDbqxcqGGSaalcqGGBbWwcqWGWbaCcqGHRaWkcqaIXaqmcqGGSaalcuWGKbazgaGbamaaBaaaleaacqaIXaqmaeqaaOGaeiyxa0LaeiykaKIaeiilaWIaeiOla4IaeiOla4IaeiOla4IaeiilaWIaeiikaGIaei4waSLafmyyaeMbayaadaWgaaWcbaGaemOta4KaeyOeI0Iaem4saSKaey4kaSIaeGymaedabeaakiabcYcaSiqbdkgaIzaagaWaaSbaaSqaaiabd6eaojabgkHiTiabdUealjabgUcaRiabigdaXaqabaGccqGGDbqxcqGGSaalcqGGBbWwcuWGJbWygaGbamaaBaaaleaacqWGobGtcqGHsislcqWGlbWscqGHRaWkcqaIXaqmaeqaaOGaeiilaWIafmizaqMbayaadaWgaaWcbaGaemOta4KaeyOeI0Iaem4saSKaey4kaSIaeGymaedabeaakiabc2faDjabcMcaPiabc2faDbaa@6E7B@ the *N*-map Γ=[([a′1,b′1],[c′1,d′1]),...,([a′K,b″1],[c′K,d″1]),...,([a″N−K+1,b″N−K+1],[c″N−K+1,d″N−K+1])]
 MathType@MTEF@5@5@+=feaafiart1ev1aaatCvAUfKttLearuWrP9MDH5MBPbIqV92AaeXatLxBI9gBaebbnrfifHhDYfgasaacH8akY=wiFfYdH8Gipec8Eeeu0xXdbba9frFj0=OqFfea0dXdd9vqai=hGuQ8kuc9pgc9s8qqaq=dirpe0xb9q8qiLsFr0=vr0=vr0dc8meaabaqaciaacaGaaeqabaqabeGadaaakeaacqqHtoWrcqGH9aqpcqGGBbWwcqGGOaakcqGGBbWwcuWGHbqygaqbamaaBaaaleaacqaIXaqmaeqaaOGaeiilaWIafmOyaiMbauaadaWgaaWcbaGaeGymaedabeaakiabc2faDjabcYcaSiabcUfaBjqbdogaJzaafaWaaSbaaSqaaiabigdaXaqabaGccqGGSaalcuWGKbazgaqbamaaBaaaleaacqaIXaqmaeqaaOGaeiyxa0LaeiykaKIaeiilaWIaeiOla4IaeiOla4IaeiOla4IaeiilaWIaeiikaGIaei4waSLafmyyaeMbauaadaWgaaWcbaGaem4saSeabeaakiabcYcaSiqbdkgaIzaagaWaaSbaaSqaaiabigdaXaqabaGccqGGDbqxcqGGSaalcqGGBbWwcuWGJbWygaqbamaaBaaaleaacqWGlbWsaeqaaOGaeiilaWIafmizaqMbayaadaWgaaWcbaGaeGymaedabeaakiabc2faDjabcMcaPiabcYcaSiabc6caUiabc6caUiabc6caUiabcYcaSiabcIcaOiabcUfaBjqbdggaHzaagaWaaSbaaSqaaiabd6eaojabgkHiTiabdUealjabgUcaRiabigdaXaqabaGccqGGSaalcuWGIbGygaGbamaaBaaaleaacqWGobGtcqGHsislcqWGlbWscqGHRaWkcqaIXaqmaeqaaOGaeiyxa0LaeiilaWIaei4waSLafm4yamMbayaadaWgaaWcbaGaemOta4KaeyOeI0Iaem4saSKaey4kaSIaeGymaedabeaakiabcYcaSiqbdsgaKzaagaWaaSbaaSqaaiabd6eaojabgkHiTiabdUealjabgUcaRiabigdaXaqabaGccqGGDbqxcqGGPaqkcqGGDbqxaaa@8518@ has score S(Γ′)+S(Γ″)
 MathType@MTEF@5@5@+=feaafiart1ev1aaatCvAUfKttLearuWrP9MDH5MBPbIqV92AaeXatLxBI9gBaebbnrfifHhDYfgasaacH8akY=wiFfYdH8Gipec8Eeeu0xXdbba9frFj0=OqFfea0dXdd9vqai=hGuQ8kuc9pgc9s8qqaq=dirpe0xb9q8qiLsFr0=vr0=vr0dc8meaabaqaciaacaGaaeqabaqabeGadaaakeaat0uy0HwzTfgDPnwy1egaryqtHrhAL1wy0L2yHvdaiqaacqWFse=ucqGGOaakcuqHtoWrgaqbaiabcMcaPiabgUcaRiab=jr8tjabcIcaOiqbfo5ahzaagaGaeiykaKcaaa@414E@, which is by definition smaller than ℳN
 MathType@MTEF@5@5@+=feaafiart1ev1aaatCvAUfKttLearuWrP9MDH5MBPbIqV92AaeXatLxBI9gBaebbnrfifHhDYfgasaacH8akY=wiFfYdH8Gipec8Eeeu0xXdbba9frFj0=OqFfea0dXdd9vqai=hGuQ8kuc9pgc9s8qqaq=dirpe0xb9q8qiLsFr0=vr0=vr0dc8meaabaqaciaacaGaaeqabaqabeGadaaakeaat0uy0HwzTfgDPnwy1egaryqtHrhAL1wy0L2yHvdaiqaacqWFZestdaahaaWcbeqaaiabd6eaobaaaaa@38E1@ (*s*, *t*). It implies that Q1≤ℳN(s,t)
 MathType@MTEF@5@5@+=feaafiart1ev1aaatCvAUfKttLearuWrP9MDH5MBPbIqV92AaeXatLxBI9gBaebbnrfifHhDYfgasaacH8akY=wiFfYdH8Gipec8Eeeu0xXdbba9frFj0=OqFfea0dXdd9vqai=hGuQ8kuc9pgc9s8qqaq=dirpe0xb9q8qiLsFr0=vr0=vr0dc8meaabaqaciaacaGaaeqabaqabeGadaaakeaat0uy0HwzTfgDPnwy1egaryqtHrhAL1wy0L2yHvdaiqaacqWFqeFudaWgaaWcbaGaeGymaedabeaakiabgsMiJkab=ntinnaaCaaaleqabaGaemOta4eaaOGaeiikaGIaem4CamNaeiilaWIaemiDaqNaeiykaKcaaa@430F@. A similar argument establishes that Q2≤ℳN(s,t)
 MathType@MTEF@5@5@+=feaafiart1ev1aaatCvAUfKttLearuWrP9MDH5MBPbIqV92AaeXatLxBI9gBaebbnrfifHhDYfgasaacH8akY=wiFfYdH8Gipec8Eeeu0xXdbba9frFj0=OqFfea0dXdd9vqai=hGuQ8kuc9pgc9s8qqaq=dirpe0xb9q8qiLsFr0=vr0=vr0dc8meaabaqaciaacaGaaeqabaqabeGadaaakeaat0uy0HwzTfgDPnwy1egaryqtHrhAL1wy0L2yHvdaiqaacqWFqeFudaWgaaWcbaGaeGOmaidabeaakiabgsMiJkab=ntinnaaCaaaleqabaGaemOta4eaaOGaeiikaGIaem4CamNaeiilaWIaemiDaqNaeiykaKcaaa@4311@ and ends the proof.

**Remark 1 ***Let s and t be two sequences, N a positive integer, and *[*D*_1_, ..., *D*_*N*_] *an optimal N-map of s over t with diagonals D*_1_, ..., *D*_*N *_*indexed following the increasing order of their s-intervals*.

*1. For all *1 ≤ *K *<*N*, [*D*_1_, ... , *D*_*K*_] *is an optimal K-map of *s[1,aK+1−1]
 MathType@MTEF@5@5@+=feaafiart1ev1aaatCvAUfKttLearuWrP9MDH5MBPbIqV92AaeXatLxBI9gBaebbnrfifHhDYfgasaacH8akY=wiFfYdH8Gipec8Eeeu0xXdbba9frFj0=OqFfea0dXdd9vqai=hGuQ8kuc9pgc9s8qqaq=dirpe0xb9q8qiLsFr0=vr0=vr0dc8meaabaqaciaacaGaaeqabaqabeGadaaakeaacqWGZbWCdaWgaaWcbaGaei4waSLaeGymaeJaeiilaWIaemyyae2aaSbaaWqaaiabdUealjabgUcaRiabigdaXaqabaWccqGHsislcqaIXaqmcqGGDbqxaeqaaaaa@38E8@*over t. Reciprocally, if *[D′1,...,D′K]
 MathType@MTEF@5@5@+=feaafiart1ev1aaatCvAUfKttLearuWrP9MDH5MBPbIqV92AaeXatLxBI9gBaebbnrfifHhDYfgasaacH8akY=wiFfYdH8Gipec8Eeeu0xXdbba9frFj0=OqFfea0dXdd9vqai=hGuQ8kuc9pgc9s8qqaq=dirpe0xb9q8qiLsFr0=vr0=vr0dc8meaabaqaciaacaGaaeqabaqabeGadaaakeaacqGGBbWwcuWGebargaqbamaaBaaaleaacqaIXaqmaeqaaOGaeiilaWIaeiOla4IaeiOla4IaeiOla4IaeiilaWIafmiraqKbauaadaWgaaWcbaGaem4saSeabeaakiabc2faDbaa@384D@*is an optimal K-map of *s[1,aK+1−1]
 MathType@MTEF@5@5@+=feaafiart1ev1aaatCvAUfKttLearuWrP9MDH5MBPbIqV92AaeXatLxBI9gBaebbnrfifHhDYfgasaacH8akY=wiFfYdH8Gipec8Eeeu0xXdbba9frFj0=OqFfea0dXdd9vqai=hGuQ8kuc9pgc9s8qqaq=dirpe0xb9q8qiLsFr0=vr0=vr0dc8meaabaqaciaacaGaaeqabaqabeGadaaakeaacqWGZbWCdaWgaaWcbaGaei4waSLaeGymaeJaeiilaWIaemyyae2aaSbaaWqaaiabdUealjabgUcaRiabigdaXaqabaWccqGHsislcqaIXaqmcqGGDbqxaeqaaaaa@38E8@*over t then *[D′1,...,D′K,DK+1,...,DN]
 MathType@MTEF@5@5@+=feaafiart1ev1aaatCvAUfKttLearuWrP9MDH5MBPbIqV92AaeXatLxBI9gBaebbnrfifHhDYfgasaacH8akY=wiFfYdH8Gipec8Eeeu0xXdbba9frFj0=OqFfea0dXdd9vqai=hGuQ8kuc9pgc9s8qqaq=dirpe0xb9q8qiLsFr0=vr0=vr0dc8meaabaqaciaacaGaaeqabaqabeGadaaakeaacqGGBbWwcuWGebargaqbamaaBaaaleaacqaIXaqmaeqaaOGaeiilaWIaeiOla4IaeiOla4IaeiOla4IaeiilaWIafmiraqKbauaadaWgaaWcbaGaem4saSeabeaakiabcYcaSiabdseaenaaBaaaleaacqWGlbWscqGHRaWkcqaIXaqmaeqaaOGaeiilaWIaeiOla4IaeiOla4IaeiOla4IaeiilaWIaemiraq0aaSbaaSqaaiabd6eaobqabaGccqGGDbqxaaa@443D@*is an optimal N-map of s over t*.

*2. For all *1 <*K *≤ *N*, [*D*_*K*_, ..., *D*_*N*_] *is an optimal *(*N *- *K *+ 1)-*map of *s[bK−1+1,|s|]
 MathType@MTEF@5@5@+=feaafiart1ev1aaatCvAUfKttLearuWrP9MDH5MBPbIqV92AaeXatLxBI9gBaebbnrfifHhDYfgasaacH8akY=wiFfYdH8Gipec8Eeeu0xXdbba9frFj0=OqFfea0dXdd9vqai=hGuQ8kuc9pgc9s8qqaq=dirpe0xb9q8qiLsFr0=vr0=vr0dc8meaabaqaciaacaGaaeqabaqabeGadaaakeaacqWGZbWCdaWgaaWcbaGaei4waSLaemOyai2aaSbaaWqaaiabdUealjabgkHiTiabigdaXaqabaWccqGHRaWkcqaIXaqmcqGGSaaldaabdaqaaiabdohaZbGaay5bSlaawIa7aiabc2faDbqabaaaaa@3C8B@*over t. Reciprocally, if *[D′K,...,D′N]
 MathType@MTEF@5@5@+=feaafiart1ev1aaatCvAUfKttLearuWrP9MDH5MBPbIqV92AaeXatLxBI9gBaebbnrfifHhDYfgasaacH8akY=wiFfYdH8Gipec8Eeeu0xXdbba9frFj0=OqFfea0dXdd9vqai=hGuQ8kuc9pgc9s8qqaq=dirpe0xb9q8qiLsFr0=vr0=vr0dc8meaabaqaciaacaGaaeqabaqabeGadaaakeaacqGGBbWwcuWGebargaqbamaaBaaaleaacqWGlbWsaeqaaOGaeiilaWIaeiOla4IaeiOla4IaeiOla4IaeiilaWIafmiraqKbauaadaWgaaWcbaGaemOta4eabeaakiabc2faDbaa@3882@*is an optimal *(*N *- *K *+ 1)-*map of *s[bK−1+1,|s|]
 MathType@MTEF@5@5@+=feaafiart1ev1aaatCvAUfKttLearuWrP9MDH5MBPbIqV92AaeXatLxBI9gBaebbnrfifHhDYfgasaacH8akY=wiFfYdH8Gipec8Eeeu0xXdbba9frFj0=OqFfea0dXdd9vqai=hGuQ8kuc9pgc9s8qqaq=dirpe0xb9q8qiLsFr0=vr0=vr0dc8meaabaqaciaacaGaaeqabaqabeGadaaakeaacqWGZbWCdaWgaaWcbaGaei4waSLaemOyai2aaSbaaWqaaiabdUealjabgkHiTiabigdaXaqabaWccqGHRaWkcqaIXaqmcqGGSaaldaabdaqaaiabdohaZbGaay5bSlaawIa7aiabc2faDbqabaaaaa@3C8B@*over t then *[D1,...,DK−1,D′K,...,D′N]
 MathType@MTEF@5@5@+=feaafiart1ev1aaatCvAUfKttLearuWrP9MDH5MBPbIqV92AaeXatLxBI9gBaebbnrfifHhDYfgasaacH8akY=wiFfYdH8Gipec8Eeeu0xXdbba9frFj0=OqFfea0dXdd9vqai=hGuQ8kuc9pgc9s8qqaq=dirpe0xb9q8qiLsFr0=vr0=vr0dc8meaabaqaciaacaGaaeqabaqabeGadaaakeaacqGGBbWwcqWGebardaWgaaWcbaGaeGymaedabeaakiabcYcaSiabc6caUiabc6caUiabc6caUiabcYcaSiabdseaenaaBaaaleaacqWGlbWscqGHsislcqaIXaqmaeqaaOGaeiilaWIafmiraqKbauaadaWgaaWcbaGaem4saSeabeaakiabcYcaSiabc6caUiabc6caUiabc6caUiabcYcaSiqbdseaezaafaWaaSbaaSqaaiabd6eaobqabaGccqGGDbqxaaa@4448@*is an optimal N-map of s over t*.

We are now able to introduce the formal algorithm **Alg_2 **which solves the problem of outputting the diagonals of an optimal global *N*-map without inversion.

Algorithm **Alg_2 **takes as inputs two sequences *s *and *t*, two positions *i *and *j *bounding a substring of *s*, and a number of parts *N*. It outputs the diagonals of an optimal *N*-map of *s*_[*i*, *j*] _over *t *ordered according to their first intervals.

**Algorithm 2 Alg_2 **(*s*, *i*, *j*, *t*, *N*)

1: **if ***N *= 0 **then**

2:   **return**;

3: **end if**

4: S_max _← -∞ ; m=⌊i+j2⌋
 MathType@MTEF@5@5@+=feaafiart1ev1aaatCvAUfKttLearuWrP9MDH5MBPbIqV92AaeXatLxBI9gBaebbnrfifHhDYfgasaacH8akY=wiFfYdH8Gipec8Eeeu0xXdbba9frFj0=OqFfea0dXdd9vqai=hGuQ8kuc9pgc9s8qqaq=dirpe0xb9q8qiLsFr0=vr0=vr0dc8meaabaqaciaacaGaaeqabaqabeGadaaakeaacqWGTbqBcqGH9aqpdaGbdaqaamaalaaabaGaemyAaKMaey4kaSIaemOAaOgabaGaeGOmaidaaaGaayj84laawUp+aaaa@38C9@ ; D′max⁡
 MathType@MTEF@5@5@+=feaafiart1ev1aaatCvAUfKttLearuWrP9MDH5MBPbIqV92AaeXatLxBI9gBaebbnrfifHhDYfgasaacH8akY=wiFfYdH8Gipec8Eeeu0xXdbba9frFj0=OqFfea0dXdd9vqai=hGuQ8kuc9pgc9s8qqaq=dirpe0xb9q8qiLsFr0=vr0=vr0dc8meaabaqaciaacaGaaeqabaqabeGadaaakeaacuqGebargaqbamaaBaaaleaacyGGTbqBcqGGHbqycqGG4baEaeqaaaaa@3219@ ← NULL ;

5: **Alg_1 **(*s*_[*i*, *m*]_, *t*, *N*, B_s_, B_l_, M_s_, M_d_) ;

6: **Alg_1 **(s[m+1,j]^,t^,N,Bs∗,Bl∗,Ms∗,Md∗)
 MathType@MTEF@5@5@+=feaafiart1ev1aaatCvAUfKttLearuWrP9MDH5MBPbIqV92AaeXatLxBI9gBaebbnrfifHhDYfgasaacH8akY=wiFfYdH8Gipec8Eeeu0xXdbba9frFj0=OqFfea0dXdd9vqai=hGuQ8kuc9pgc9s8qqaq=dirpe0xb9q8qiLsFr0=vr0=vr0dc8meaabaqaciaacaGaaeqabaqabeGadaaakeaacqGGOaakdaqiaaqaaiabdohaZnaaBaaaleaacqGGBbWwcqWGTbqBcqGHRaWkcqaIXaqmcqGGSaalcqWGQbGAcqGGDbqxaeqaaaGccaGLcmaacqGGSaalcuWG0baDgaqcaiabcYcaSiabd6eaojabcYcaSiabbkeacnaaDaaaleaacqqGZbWCaeaacqGHxiIkaaGccqGGSaalcqqGcbGqdaqhaaWcbaGaeeiBaWgabaGaey4fIOcaaOGaeiilaWIaeeyta00aa0baaSqaaiabbohaZbqaaiabgEHiQaaakiabcYcaSiabb2eannaaDaaaleaacqqGKbazaeaacqGHxiIkaaGccqGGPaqkaaa@4F15@ ;         \* *Loop *Q1
 MathType@MTEF@5@5@+=feaafiart1ev1aaatCvAUfKttLearuWrP9MDH5MBPbIqV92AaeXatLxBI9gBaebbnrfifHhDYfgasaacH8akY=wiFfYdH8Gipec8Eeeu0xXdbba9frFj0=OqFfea0dXdd9vqai=hGuQ8kuc9pgc9s8qqaq=dirpe0xb9q8qiLsFr0=vr0=vr0dc8meaabaqaciaacaGaaeqabaqabeGadaaakeaat0uy0HwzTfgDPnwy1egaryqtHrhAL1wy0L2yHvdaiqaacqWFqeFudaWgaaWcbaGaeGymaedabeaaaaa@395C@ *\

7: **for ***K *← 1 **to *N *do**

8:   *L *← *N *- *K *+ 1 ;

9:   **for ***p *← 1 **to **(|*t*| - 1) **do**

10:      *q *← |*t*| - *p *;

11:      **if **(B_s[*p*, *K*] _+ Bs[q,L]∗
 MathType@MTEF@5@5@+=feaafiart1ev1aaatCvAUfKttLearuWrP9MDH5MBPbIqV92AaeXatLxBI9gBaebbnrfifHhDYfgasaacH8akY=wiFfYdH8Gipec8Eeeu0xXdbba9frFj0=OqFfea0dXdd9vqai=hGuQ8kuc9pgc9s8qqaq=dirpe0xb9q8qiLsFr0=vr0=vr0dc8meaabaqaciaacaGaaeqabaqabeGadaaakeaacqqGcbGqdaqhaaWcbaGaee4CamNaei4waSLaemyCaeNaeiilaWIaemitaWKaeiyxa0fabaGaey4fIOcaaaaa@362C@) > S_max _**then**

12:         S_max _← B_s[*p, K*] _+ Bs[q,L]∗
 MathType@MTEF@5@5@+=feaafiart1ev1aaatCvAUfKttLearuWrP9MDH5MBPbIqV92AaeXatLxBI9gBaebbnrfifHhDYfgasaacH8akY=wiFfYdH8Gipec8Eeeu0xXdbba9frFj0=OqFfea0dXdd9vqai=hGuQ8kuc9pgc9s8qqaq=dirpe0xb9q8qiLsFr0=vr0=vr0dc8meaabaqaciaacaGaaeqabaqabeGadaaakeaacqqGcbGqdaqhaaWcbaGaee4CamNaei4waSLaemyCaeNaeiilaWIaemitaWKaeiyxa0fabaGaey4fIOcaaaaa@362C@ ;

13:         D_max _← ([*m *- B_l[*p, K*] _+ 1, *m *+ Bl[q,L]∗
 MathType@MTEF@5@5@+=feaafiart1ev1aaatCvAUfKttLearuWrP9MDH5MBPbIqV92AaeXatLxBI9gBaebbnrfifHhDYfgasaacH8akY=wiFfYdH8Gipec8Eeeu0xXdbba9frFj0=OqFfea0dXdd9vqai=hGuQ8kuc9pgc9s8qqaq=dirpe0xb9q8qiLsFr0=vr0=vr0dc8meaabaqaciaacaGaaeqabaqabeGadaaakeaacqqGcbGqdaqhaaWcbaGaeeiBaWMaei4waSLaemyCaeNaeiilaWIaemitaWKaeiyxa0fabaGaey4fIOcaaaaa@361E@], [*p *- B_l[*p, K*] _+ l, *p *+ Bl[q,L]∗
 MathType@MTEF@5@5@+=feaafiart1ev1aaatCvAUfKttLearuWrP9MDH5MBPbIqV92AaeXatLxBI9gBaebbnrfifHhDYfgasaacH8akY=wiFfYdH8Gipec8Eeeu0xXdbba9frFj0=OqFfea0dXdd9vqai=hGuQ8kuc9pgc9s8qqaq=dirpe0xb9q8qiLsFr0=vr0=vr0dc8meaabaqaciaacaGaaeqabaqabeGadaaakeaacqqGcbGqdaqhaaWcbaGaeeiBaWMaei4waSLaemyCaeNaeiilaWIaemitaWKaeiyxa0fabaGaey4fIOcaaaaa@361E@]) ;

14:         *N*_L _← *K *- 1 ; *N*_R _← *L *- 1 ; *j*_L _← *m *- B_l[*p*, *K*] _; *i*_R _← *m *+ Bl[q,L]∗
 MathType@MTEF@5@5@+=feaafiart1ev1aaatCvAUfKttLearuWrP9MDH5MBPbIqV92AaeXatLxBI9gBaebbnrfifHhDYfgasaacH8akY=wiFfYdH8Gipec8Eeeu0xXdbba9frFj0=OqFfea0dXdd9vqai=hGuQ8kuc9pgc9s8qqaq=dirpe0xb9q8qiLsFr0=vr0=vr0dc8meaabaqaciaacaGaaeqabaqabeGadaaakeaacqqGcbGqdaqhaaWcbaGaeeiBaWMaei4waSLaemyCaeNaeiilaWIaemitaWKaeiyxa0fabaGaey4fIOcaaaaa@361E@ + 1;

15:      **end if**

16:   **end for**

17: **end for**         \* *Loop *Q2
 MathType@MTEF@5@5@+=feaafiart1ev1aaatCvAUfKttLearuWrP9MDH5MBPbIqV92AaeXatLxBI9gBaebbnrfifHhDYfgasaacH8akY=wiFfYdH8Gipec8Eeeu0xXdbba9frFj0=OqFfea0dXdd9vqai=hGuQ8kuc9pgc9s8qqaq=dirpe0xb9q8qiLsFr0=vr0=vr0dc8meaabaqaciaacaGaaeqabaqabeGadaaakeaat0uy0HwzTfgDPnwy1egaryqtHrhAL1wy0L2yHvdaiqaacqWFqeFudaWgaaWcbaGaeGOmaidabeaaaaa@395E@ *\

18: **for ***K *← 0 **to ***N ***do**

19:   *L *← *N *- *K *;

20:   **if **(M_s[*K*] _+ Ms[L]∗
 MathType@MTEF@5@5@+=feaafiart1ev1aaatCvAUfKttLearuWrP9MDH5MBPbIqV92AaeXatLxBI9gBaebbnrfifHhDYfgasaacH8akY=wiFfYdH8Gipec8Eeeu0xXdbba9frFj0=OqFfea0dXdd9vqai=hGuQ8kuc9pgc9s8qqaq=dirpe0xb9q8qiLsFr0=vr0=vr0dc8meaabaqaciaacaGaaeqabaqabeGadaaakeaacqqGnbqtdaqhaaWcbaGaee4CamNaei4waSLaemitaWKaeiyxa0fabaGaey4fIOcaaaaa@33F7@) > S_max _**then**

21:      S_max _← M_s[*K*] _+ Ms[L]∗
 MathType@MTEF@5@5@+=feaafiart1ev1aaatCvAUfKttLearuWrP9MDH5MBPbIqV92AaeXatLxBI9gBaebbnrfifHhDYfgasaacH8akY=wiFfYdH8Gipec8Eeeu0xXdbba9frFj0=OqFfea0dXdd9vqai=hGuQ8kuc9pgc9s8qqaq=dirpe0xb9q8qiLsFr0=vr0=vr0dc8meaabaqaciaacaGaaeqabaqabeGadaaakeaacqqGnbqtdaqhaaWcbaGaee4CamNaei4waSLaemitaWKaeiyxa0fabaGaey4fIOcaaaaa@33F7@

22:      **if ***K *> 0 **and **M_d[*K*] _≠ NULL **then**

23:         ([*a*, *b*], [*c*, *d*]) ← M_d[*K*] _; D_max _← ([*a *+ *i *- 1, *b *+ *i *- 1], [*c*, *d*]) ;

24:         *N*_L _← *K *- 1 ; *j*_L _← *a *+ *i *- 2 ;

25:      **else**

26:         D_max _← NULL ; *N*_L _← 0 ;

27:      **end if**

28:      **if ***L *> 0 **and **Md[L]∗
 MathType@MTEF@5@5@+=feaafiart1ev1aaatCvAUfKttLearuWrP9MDH5MBPbIqV92AaeXatLxBI9gBaebbnrfifHhDYfgasaacH8akY=wiFfYdH8Gipec8Eeeu0xXdbba9frFj0=OqFfea0dXdd9vqai=hGuQ8kuc9pgc9s8qqaq=dirpe0xb9q8qiLsFr0=vr0=vr0dc8meaabaqaciaacaGaaeqabaqabeGadaaakeaacqqGnbqtdaqhaaWcbaGaeeizaqMaei4waSLaemitaWKaeiyxa0fabaGaey4fIOcaaaaa@33D9@ ≠ NULL **then**

29:         ([*a*, *b*], [*c*, *d*]) ← Md[L]∗
 MathType@MTEF@5@5@+=feaafiart1ev1aaatCvAUfKttLearuWrP9MDH5MBPbIqV92AaeXatLxBI9gBaebbnrfifHhDYfgasaacH8akY=wiFfYdH8Gipec8Eeeu0xXdbba9frFj0=OqFfea0dXdd9vqai=hGuQ8kuc9pgc9s8qqaq=dirpe0xb9q8qiLsFr0=vr0=vr0dc8meaabaqaciaacaGaaeqabaqabeGadaaakeaacqqGnbqtdaqhaaWcbaGaeeizaqMaei4waSLaemitaWKaeiyxa0fabaGaey4fIOcaaaaa@33D9@ ; D′max⁡
 MathType@MTEF@5@5@+=feaafiart1ev1aaatCvAUfKttLearuWrP9MDH5MBPbIqV92AaeXatLxBI9gBaebbnrfifHhDYfgasaacH8akY=wiFfYdH8Gipec8Eeeu0xXdbba9frFj0=OqFfea0dXdd9vqai=hGuQ8kuc9pgc9s8qqaq=dirpe0xb9q8qiLsFr0=vr0=vr0dc8meaabaqaciaacaGaaeqabaqabeGadaaakeaacuqGebargaqbamaaBaaaleaacyGGTbqBcqGGHbqycqGG4baEaeqaaaaa@3219@ ← ([*a *+ *m*, *b *+ *m*], [*c*, *d*]) ;

30:         *N*_R _← *L *- 1 ; *i*_R _← *b *+ *m *+ 1 ;

31:      **else**

32:         D′max⁡
 MathType@MTEF@5@5@+=feaafiart1ev1aaatCvAUfKttLearuWrP9MDH5MBPbIqV92AaeXatLxBI9gBaebbnrfifHhDYfgasaacH8akY=wiFfYdH8Gipec8Eeeu0xXdbba9frFj0=OqFfea0dXdd9vqai=hGuQ8kuc9pgc9s8qqaq=dirpe0xb9q8qiLsFr0=vr0=vr0dc8meaabaqaciaacaGaaeqabaqabeGadaaakeaacuqGebargaqbamaaBaaaleaacyGGTbqBcqGGHbqycqGG4baEaeqaaaaa@3219@ ← NULL ; *N*_R _← 0 ;

33:      **end if**

34:   **end if**

35: **end for**

36: **Alg_2 **(*s*, *i*, *j*_L_, *t*, *N*_L_) ;

37: **Output **(D_max_) ; **output **(D′max⁡
 MathType@MTEF@5@5@+=feaafiart1ev1aaatCvAUfKttLearuWrP9MDH5MBPbIqV92AaeXatLxBI9gBaebbnrfifHhDYfgasaacH8akY=wiFfYdH8Gipec8Eeeu0xXdbba9frFj0=OqFfea0dXdd9vqai=hGuQ8kuc9pgc9s8qqaq=dirpe0xb9q8qiLsFr0=vr0=vr0dc8meaabaqaciaacaGaaeqabaqabeGadaaakeaacuqGebargaqbamaaBaaaleaacyGGTbqBcqGGHbqycqGG4baEaeqaaaaa@3219@) ;

38: **Alg_2 **(*s*, *i*_R_, *j*, *t*, *N*_R_) ;

#### Correctness of Algorithm Alg_2

Let us consider Best and Best¯
 MathType@MTEF@5@5@+=feaafiart1ev1aaatCvAUfKttLearuWrP9MDH5MBPbIqV92AaeXatLxBI9gBaebbnrfifHhDYfgasaacH8akY=wiFfYdH8Gipec8Eeeu0xXdbba9frFj0=OqFfea0dXdd9vqai=hGuQ8kuc9pgc9s8qqaq=dirpe0xb9q8qiLsFr0=vr0=vr0dc8meaabaqaciaacaGaaeqabaqabeGadaaakeaadaqdaaqaaiabbkeacjabbwgaLjabbohaZjabbsha0baaaaa@31F5@ defined for *s*_[*i*, *j*] _as follows. For all *r *such that *i *≤ *r *≤ *j *and *v *such that 1 ≤ *v *≤ |*t*|, Best_[*r*, *v*, *K*] _is the maximal score obtained by a *K*-map of *s*_[*i*, *r*] _over *t*_[1, *v*] _ending at (*r, v*). Analogously, Best¯[r,v,K]
 MathType@MTEF@5@5@+=feaafiart1ev1aaatCvAUfKttLearuWrP9MDH5MBPbIqV92AaeXatLxBI9gBaebbnrfifHhDYfgasaacH8akY=wiFfYdH8Gipec8Eeeu0xXdbba9frFj0=OqFfea0dXdd9vqai=hGuQ8kuc9pgc9s8qqaq=dirpe0xb9q8qiLsFr0=vr0=vr0dc8meaabaqaciaacaGaaeqabaqabeGadaaakeaadaqdaaqaaiabbkeacjabbwgaLjabbohaZjabbsha0baadaWgaaWcbaGaei4waSLaemOCaiNaeiilaWIaemODayNaeiilaWIaem4saSKaeiyxa0fabeaaaaa@3A62@ is the maximal score obtained by a *K*-map of *s*_[*r*, *j*] _over *t*_[*v*, |*t*|] _starting at (*r*, *v*). For all positions *p *of *t *and all 1 ≤ *K *≤ *N*, we have B_s[*p, K*] _= Best_[*m*, *p*, *K*]_, Bs[|t|−p,K]∗=Best¯[m+1,p+1,K]
 MathType@MTEF@5@5@+=feaafiart1ev1aaatCvAUfKttLearuWrP9MDH5MBPbIqV92AaeXatLxBI9gBaebbnrfifHhDYfgasaacH8akY=wiFfYdH8Gipec8Eeeu0xXdbba9frFj0=OqFfea0dXdd9vqai=hGuQ8kuc9pgc9s8qqaq=dirpe0xb9q8qiLsFr0=vr0=vr0dc8meaabaqaciaacaGaaeqabaqabeGadaaakeaacqqGcbGqdaqhaaWcbaGaee4CamNaei4waSLaeiiFaWNaemiDaqNaeiiFaWNaeyOeI0IaemiCaaNaeiilaWIaem4saSKaeiyxa0fabaGaey4fIOcaaOGaeyypa0Zaa0aaaeaacqqGcbGqcqqGLbqzcqqGZbWCcqqG0baDaaWaaSbaaSqaaiabcUfaBjabd2gaTjabgUcaRiabigdaXiabcYcaSiabdchaWjabgUcaRiabigdaXiabcYcaSiabdUealjabc2faDbqabaaaaa@4DDA@ (since it is obtained from s[m+1,j]^
 MathType@MTEF@5@5@+=feaafiart1ev1aaatCvAUfKttLearuWrP9MDH5MBPbIqV92AaeXatLxBI9gBaebbnrfifHhDYfgasaacH8akY=wiFfYdH8Gipec8Eeeu0xXdbba9frFj0=OqFfea0dXdd9vqai=hGuQ8kuc9pgc9s8qqaq=dirpe0xb9q8qiLsFr0=vr0=vr0dc8meaabaqaciaacaGaaeqabaqabeGadaaakeaadaqiaaqaaiabdohaZnaaBaaaleaacqGGBbWwcqWGTbqBcqGHRaWkcqaIXaqmcqGGSaalcqWGQbGAcqGGDbqxaeqaaaGccaGLcmaaaaa@3705@), Ms[K]=ℳK(s[i,m],t)
 MathType@MTEF@5@5@+=feaafiart1ev1aaatCvAUfKttLearuWrP9MDH5MBPbIqV92AaeXatLxBI9gBaebbnrfifHhDYfgasaacH8akY=wiFfYdH8Gipec8Eeeu0xXdbba9frFj0=OqFfea0dXdd9vqai=hGuQ8kuc9pgc9s8qqaq=dirpe0xb9q8qiLsFr0=vr0=vr0dc8meaabaqaciaacaGaaeqabaqabeGadaaakeaacqqGnbqtdaWgaaWcbaGaee4CamNaei4waSLaem4saSKaeiyxa0fabeaakiabg2da9mrtHrhAL1wy0L2yHvtyaeHbnfgDOvwBHrxAJfwnaGabaiab=ntinnaaCaaaleqabaGaem4saSeaaOGaeiikaGIaem4Cam3aaSbaaSqaaiabcUfaBjabdMgaPjabcYcaSiabd2gaTjabc2faDjabcYcaSaqabaGccqWG0baDcqGGPaqkaaa@4C14@ and Ms[K]∗=ℳK(sm+1,j],t)
 MathType@MTEF@5@5@+=feaafiart1ev1aaatCvAUfKttLearuWrP9MDH5MBPbIqV92AaeXatLxBI9gBaebbnrfifHhDYfgasaacH8akY=wiFfYdH8Gipec8Eeeu0xXdbba9frFj0=OqFfea0dXdd9vqai=hGuQ8kuc9pgc9s8qqaq=dirpe0xb9q8qiLsFr0=vr0=vr0dc8meaabaqaciaacaGaaeqabaqabeGadaaakeaacqqGnbqtdaqhaaWcbaGaee4CamNaei4waSLaem4saSKaeiyxa0fabaGaey4fIOcaaOGaeyypa0ZenfgDOvwBHrxAJfwnHbqeg0uy0HwzTfgDPnwy1aaceaGae83mH00aaWbaaSqabeaacqWGlbWsaaGccqGGOaakcqWGZbWCdaWgaaWcbaGaemyBa0Maey4kaSIaeGymaeJaeiilaWIaemOAaOMaeiyxa0fabeaakiabcYcaSiabdsha0jabcMcaPaaa@4D9A@. Following the notations of Lemma 1, "Loop Q1
 MathType@MTEF@5@5@+=feaafiart1ev1aaatCvAUfKttLearuWrP9MDH5MBPbIqV92AaeXatLxBI9gBaebbnrfifHhDYfgasaacH8akY=wiFfYdH8Gipec8Eeeu0xXdbba9frFj0=OqFfea0dXdd9vqai=hGuQ8kuc9pgc9s8qqaq=dirpe0xb9q8qiLsFr0=vr0=vr0dc8meaabaqaciaacaGaaeqabaqabeGadaaakeaat0uy0HwzTfgDPnwy1egaryqtHrhAL1wy0L2yHvdaiqaacqWFqeFudaWgaaWcbaGaeGymaedabeaaaaa@395C@" (*resp*. "Loop Q2
 MathType@MTEF@5@5@+=feaafiart1ev1aaatCvAUfKttLearuWrP9MDH5MBPbIqV92AaeXatLxBI9gBaebbnrfifHhDYfgasaacH8akY=wiFfYdH8Gipec8Eeeu0xXdbba9frFj0=OqFfea0dXdd9vqai=hGuQ8kuc9pgc9s8qqaq=dirpe0xb9q8qiLsFr0=vr0=vr0dc8meaabaqaciaacaGaaeqabaqabeGadaaakeaat0uy0HwzTfgDPnwy1egaryqtHrhAL1wy0L2yHvdaiqaacqWFqeFudaWgaaWcbaGaeGOmaidabeaaaaa@395E@") parses the quantities maximized by Q1
 MathType@MTEF@5@5@+=feaafiart1ev1aaatCvAUfKttLearuWrP9MDH5MBPbIqV92AaeXatLxBI9gBaebbnrfifHhDYfgasaacH8akY=wiFfYdH8Gipec8Eeeu0xXdbba9frFj0=OqFfea0dXdd9vqai=hGuQ8kuc9pgc9s8qqaq=dirpe0xb9q8qiLsFr0=vr0=vr0dc8meaabaqaciaacaGaaeqabaqabeGadaaakeaat0uy0HwzTfgDPnwy1egaryqtHrhAL1wy0L2yHvdaiqaacqWFqeFudaWgaaWcbaGaeGymaedabeaaaaa@395C@ (*resp*. by Q2
 MathType@MTEF@5@5@+=feaafiart1ev1aaatCvAUfKttLearuWrP9MDH5MBPbIqV92AaeXatLxBI9gBaebbnrfifHhDYfgasaacH8akY=wiFfYdH8Gipec8Eeeu0xXdbba9frFj0=OqFfea0dXdd9vqai=hGuQ8kuc9pgc9s8qqaq=dirpe0xb9q8qiLsFr0=vr0=vr0dc8meaabaqaciaacaGaaeqabaqabeGadaaakeaat0uy0HwzTfgDPnwy1egaryqtHrhAL1wy0L2yHvdaiqaacqWFqeFudaWgaaWcbaGaeGOmaidabeaaaaa@395E@). Thus, Lemma 1 ensures that ℳN(s[i,j],t)
 MathType@MTEF@5@5@+=feaafiart1ev1aaatCvAUfKttLearuWrP9MDH5MBPbIqV92AaeXatLxBI9gBaebbnrfifHhDYfgasaacH8akY=wiFfYdH8Gipec8Eeeu0xXdbba9frFj0=OqFfea0dXdd9vqai=hGuQ8kuc9pgc9s8qqaq=dirpe0xb9q8qiLsFr0=vr0=vr0dc8meaabaqaciaacaGaaeqabaqabeGadaaakeaat0uy0HwzTfgDPnwy1egaryqtHrhAL1wy0L2yHvdaiqaacqWFZestdaahaaWcbeqaaiabd6eaobaakiabcIcaOiabdohaZnaaBaaaleaacqGGBbWwcqWGPbqAcqGGSaalcqWGQbGAcqGGDbqxaeqaaOGaeiilaWIaemiDaqNaeiykaKcaaa@44AB@ is stored in the variable S_max _after the execution of these two loops. If the maximum is reached in "Loop Q1
 MathType@MTEF@5@5@+=feaafiart1ev1aaatCvAUfKttLearuWrP9MDH5MBPbIqV92AaeXatLxBI9gBaebbnrfifHhDYfgasaacH8akY=wiFfYdH8Gipec8Eeeu0xXdbba9frFj0=OqFfea0dXdd9vqai=hGuQ8kuc9pgc9s8qqaq=dirpe0xb9q8qiLsFr0=vr0=vr0dc8meaabaqaciaacaGaaeqabaqabeGadaaakeaat0uy0HwzTfgDPnwy1egaryqtHrhAL1wy0L2yHvdaiqaacqWFqeFudaWgaaWcbaGaeGymaedabeaaaaa@395C@", the variable D′max⁡
 MathType@MTEF@5@5@+=feaafiart1ev1aaatCvAUfKttLearuWrP9MDH5MBPbIqV92AaeXatLxBI9gBaebbnrfifHhDYfgasaacH8akY=wiFfYdH8Gipec8Eeeu0xXdbba9frFj0=OqFfea0dXdd9vqai=hGuQ8kuc9pgc9s8qqaq=dirpe0xb9q8qiLsFr0=vr0=vr0dc8meaabaqaciaacaGaaeqabaqabeGadaaakeaacuqGebargaqbamaaBaaaleaacyGGTbqBcqGGHbqycqGG4baEaeqaaaaa@3219@ is NULL and the variable D_max _contains the diagonal including *m*, let us say the *K*^th^, of a *N*-map with score ℳN(s[i,j],t)
 MathType@MTEF@5@5@+=feaafiart1ev1aaatCvAUfKttLearuWrP9MDH5MBPbIqV92AaeXatLxBI9gBaebbnrfifHhDYfgasaacH8akY=wiFfYdH8Gipec8Eeeu0xXdbba9frFj0=OqFfea0dXdd9vqai=hGuQ8kuc9pgc9s8qqaq=dirpe0xb9q8qiLsFr0=vr0=vr0dc8meaabaqaciaacaGaaeqabaqabeGadaaakeaat0uy0HwzTfgDPnwy1egaryqtHrhAL1wy0L2yHvdaiqaacqWFZestdaahaaWcbeqaaiabd6eaobaakiabcIcaOiabdohaZnaaBaaaleaacqGGBbWwcqWGPbqAcqGGSaalcqWGQbGAcqGGDbqxaeqaaOGaeiilaWIaemiDaqNaeiykaKcaaa@44AB@. Remark 1 allows us to output the *K*^th ^diagonal ([*a*_*K*_, *b*_*K*_], [*c*_*K*_, *d*_*K*_]) and to compute recursively an optimal (*K *- 1)-map of s[i,aK−1]
 MathType@MTEF@5@5@+=feaafiart1ev1aaatCvAUfKttLearuWrP9MDH5MBPbIqV92AaeXatLxBI9gBaebbnrfifHhDYfgasaacH8akY=wiFfYdH8Gipec8Eeeu0xXdbba9frFj0=OqFfea0dXdd9vqai=hGuQ8kuc9pgc9s8qqaq=dirpe0xb9q8qiLsFr0=vr0=vr0dc8meaabaqaciaacaGaaeqabaqabeGadaaakeaacqWGZbWCdaWgaaWcbaGaei4waSLaemyAaKMaeiilaWIaemyyae2aaSbaaWqaaiabdUealbqabaWccqGHsislcqaIXaqmcqGGDbqxaeqaaaaa@3781@ over *t*, and a (*N *- *K*)-map of s[bK+1,j]
 MathType@MTEF@5@5@+=feaafiart1ev1aaatCvAUfKttLearuWrP9MDH5MBPbIqV92AaeXatLxBI9gBaebbnrfifHhDYfgasaacH8akY=wiFfYdH8Gipec8Eeeu0xXdbba9frFj0=OqFfea0dXdd9vqai=hGuQ8kuc9pgc9s8qqaq=dirpe0xb9q8qiLsFr0=vr0=vr0dc8meaabaqaciaacaGaaeqabaqabeGadaaakeaacqWGZbWCdaWgaaWcbaGaei4waSLaemOyai2aaSbaaWqaaiabdUealbqabaWccqGHRaWkcqaIXaqmcqGGSaalcqWGQbGAcqGGDbqxaeqaaaaa@377A@ over *t*.

If the maximum is reached in "Loop Q2
 MathType@MTEF@5@5@+=feaafiart1ev1aaatCvAUfKttLearuWrP9MDH5MBPbIqV92AaeXatLxBI9gBaebbnrfifHhDYfgasaacH8akY=wiFfYdH8Gipec8Eeeu0xXdbba9frFj0=OqFfea0dXdd9vqai=hGuQ8kuc9pgc9s8qqaq=dirpe0xb9q8qiLsFr0=vr0=vr0dc8meaabaqaciaacaGaaeqabaqabeGadaaakeaat0uy0HwzTfgDPnwy1egaryqtHrhAL1wy0L2yHvdaiqaacqWFqeFudaWgaaWcbaGaeGOmaidabeaaaaa@395E@", the variables D_max _and D′max⁡
 MathType@MTEF@5@5@+=feaafiart1ev1aaatCvAUfKttLearuWrP9MDH5MBPbIqV92AaeXatLxBI9gBaebbnrfifHhDYfgasaacH8akY=wiFfYdH8Gipec8Eeeu0xXdbba9frFj0=OqFfea0dXdd9vqai=hGuQ8kuc9pgc9s8qqaq=dirpe0xb9q8qiLsFr0=vr0=vr0dc8meaabaqaciaacaGaaeqabaqabeGadaaakeaacuqGebargaqbamaaBaaaleaacyGGTbqBcqGGHbqycqGG4baEaeqaaaaa@3219@ contain the two diagonals on both sides of position *m *of a *N*-map with score ℳN(s[i,j],t)
 MathType@MTEF@5@5@+=feaafiart1ev1aaatCvAUfKttLearuWrP9MDH5MBPbIqV92AaeXatLxBI9gBaebbnrfifHhDYfgasaacH8akY=wiFfYdH8Gipec8Eeeu0xXdbba9frFj0=OqFfea0dXdd9vqai=hGuQ8kuc9pgc9s8qqaq=dirpe0xb9q8qiLsFr0=vr0=vr0dc8meaabaqaciaacaGaaeqabaqabeGadaaakeaat0uy0HwzTfgDPnwy1egaryqtHrhAL1wy0L2yHvdaiqaacqWFZestdaahaaWcbeqaaiabd6eaobaakiabcIcaOiabdohaZnaaBaaaleaacqGGBbWwcqWGPbqAcqGGSaalcqWGQbGAcqGGDbqxaeqaaOGaeiilaWIaemiDaqNaeiykaKcaaa@44AB@. The diagonal D_max _(*resp*. D′max⁡
 MathType@MTEF@5@5@+=feaafiart1ev1aaatCvAUfKttLearuWrP9MDH5MBPbIqV92AaeXatLxBI9gBaebbnrfifHhDYfgasaacH8akY=wiFfYdH8Gipec8Eeeu0xXdbba9frFj0=OqFfea0dXdd9vqai=hGuQ8kuc9pgc9s8qqaq=dirpe0xb9q8qiLsFr0=vr0=vr0dc8meaabaqaciaacaGaaeqabaqabeGadaaakeaacuqGebargaqbamaaBaaaleaacyGGTbqBcqGGHbqycqGG4baEaeqaaaaa@3219@) is possibly NULL – and not outputted – if *m *is smaller than the first position (*resp*. greater than the last position) of the *N*-map. Applying again Remark 1 leads to the correctness of the algorithm.

#### Time and space analysis of Algorithm Alg_2

Let us consider the recursion tree of an execution of **Alg_2 **which outputs an optimal *N*-map of *s *over *t*. The root of this tree is the initial call to **Alg_2 **with the parameters (*s*, 1, |*s*|, *t*, *N*), its two children are the two recursive calls in lines 36 and 38, and so on. The depth level of recursion of the initial call/root is 0. The depth level of another call is recursively defined as the incremented depth level of its direct ancestor. Before the two recursive calls at lines 36 and 38, the execution time of a call to **Alg_2 **with the parameters (*s*, *i*, *j*, *t*, *N*) is bounded by *c *× (*j *- *i *+ 1) × |*t*| × *N*, for a constant *c*. Time is spent essentially in the two calls to **Alg_1 **at lines 5 and 6. The two recursive calls are done with the parameters (*s*, *i*, *j*_L_, *t*, *N*_L_) and (*s*, *i*_R_, *j*, *t*, *N*_R_) where:

• (jL−i+1)≤j−i+12 and (j−iR+1)≤j−i+12
 MathType@MTEF@5@5@+=feaafiart1ev1aaatCvAUfKttLearuWrP9MDH5MBPbIqV92AaeXatLxBI9gBaebbnrfifHhDYfgasaacH8akY=wiFfYdH8Gipec8Eeeu0xXdbba9frFj0=OqFfea0dXdd9vqai=hGuQ8kuc9pgc9s8qqaq=dirpe0xb9q8qiLsFr0=vr0=vr0dc8meaabaqaciaacaGaaeqabaqabeGadaaakeaacqGGOaakcqWGQbGAdaWgaaWcbaGaeeitaWeabeaakiabgkHiTiabdMgaPjabgUcaRiabigdaXiabcMcaPiabgsMiJoaalaaabaGaemOAaOMaeyOeI0IaemyAaKMaey4kaSIaeGymaedabaGaeGOmaidaaiabbccaGiabbggaHjabb6gaUjabbsgaKjabbccaGiabcIcaOiabdQgaQjabgkHiTiabdMgaPnaaBaaaleaacqqGsbGuaeqaaOGaey4kaSIaeGymaeJaeiykaKIaeyizIm6aaSaaaeaacqWGQbGAcqGHsislcqWGPbqAcqGHRaWkcqaIXaqmaeaacqaIYaGmaaaaaa@5399@

• *N*_L _+ *N*_R _≤ *N *- 1

Let us remark that because of the possibly unbalanced repartition of *N *into *N*_L _and *N*_R _between the subcalls, the Master Theorem [11], generally used to evaluate complexity of divide and conquer algorithms, cannot be applied to prove the desired time complexity.

Since the initial call is done with the parameters (*s*, 1, |*s*|, *t*, *N*), the following assertions can be proved by induction over the depth level of recursion.

• From Inequalities (1), the length of the substring of *s *bounded by the two parameters "positions" in a call of depth level *d *is smaller than |s|2d
 MathType@MTEF@5@5@+=feaafiart1ev1aaatCvAUfKttLearuWrP9MDH5MBPbIqV92AaeXatLxBI9gBaebbnrfifHhDYfgasaacH8akY=wiFfYdH8Gipec8Eeeu0xXdbba9frFj0=OqFfea0dXdd9vqai=hGuQ8kuc9pgc9s8qqaq=dirpe0xb9q8qiLsFr0=vr0=vr0dc8meaabaqaciaacaGaaeqabaqabeGadaaakeaadaWcaaqaamaaemaabaGaem4CamhacaGLhWUaayjcSdaabaGaeGOmaiZaaWbaaSqabeaacqWGKbazaaaaaaaa@33BD@.

• From Inequality (2), the sum of the parameters "number of parts" of all the calls of depth level *d *is smaller than (*N *- *d*).

Thus, the total time spent at a level of recursion *d *is smaller than *c *× |s|2d
 MathType@MTEF@5@5@+=feaafiart1ev1aaatCvAUfKttLearuWrP9MDH5MBPbIqV92AaeXatLxBI9gBaebbnrfifHhDYfgasaacH8akY=wiFfYdH8Gipec8Eeeu0xXdbba9frFj0=OqFfea0dXdd9vqai=hGuQ8kuc9pgc9s8qqaq=dirpe0xb9q8qiLsFr0=vr0=vr0dc8meaabaqaciaacaGaaeqabaqabeGadaaakeaadaWcaaqaamaaemaabaGaem4CamhacaGLhWUaayjcSdaabaGaeGOmaiZaaWbaaSqabeaacqWGKbazaaaaaaaa@33BD@ × |*t*| × *N*. By summing over all the possible levels (at most *N *levels), it comes that the total (including all the recursive subcalls) execution time of a call to **Alg_2 **with the parameters (*s*, 1, |*s*|, *t*, *N*) is smaller than 2 × *c *× |*s*| × |*t*| × *N*. This ends the time analysis.

The analysis of the memory space complexity is straightforward: each call needs only *O *(|*t*| × *N*) of local storage space to run; the sequences are stored once in *O *(|*s*| + |*t*|) and, from Inequality (2), there are at most *N *recursive calls to **Alg_2**.

**Theorem 2 ***Algorithm ***Alg_2 ***outputs the diagonals of an optimal N-map of a sequence s over a sequence t in time O *(|*s*| × |*t*| × *N*) *using O *(|*s*| + |*t*| × *N*) *memory space*.

The algorithm taking into account inversions follows the same general outline with additional and symmetrical dynamic programming variables for "reverse diagonals".

A similar idea can be used to compute an optimal alignment with a fixed number *N *of gaps in *O *(|*s*| × |*t*| × *N*) time complexity using *O *(|*s*| + |*t*| × *N*) memory space. It improves the "SANK_AL" algorithm described in [9], which needs *O *(|*s*| × |*t*| × *N*) memory space.

## 3 Choice of the number of parts

Given two sequences *s *and *t*, the score of an optimal *N*-map of *s *over *t *increases with *N*. The maximum of the optimal scores is reached at most with *N *= |*s*| and the corresponding maps generally do not make sense. Some *a priori *knowledge could help us to decide whether the increase of the score between the *K*-and the (*K *+ 1)-map deserves to consider an extra diagonal, for instance by introducing a penalty growing linearly with the number of parts.

Without such *a priori *knowledge, a natural choice is to consider the most significant optimal *N*-map: here the one which minimizes the probability of observing an optimal score greater than ℳN
 MathType@MTEF@5@5@+=feaafiart1ev1aaatCvAUfKttLearuWrP9MDH5MBPbIqV92AaeXatLxBI9gBaebbnrfifHhDYfgasaacH8akY=wiFfYdH8Gipec8Eeeu0xXdbba9frFj0=OqFfea0dXdd9vqai=hGuQ8kuc9pgc9s8qqaq=dirpe0xb9q8qiLsFr0=vr0=vr0dc8meaabaqaciaacaGaaeqabaqabeGadaaakeaat0uy0HwzTfgDPnwy1egaryqtHrhAL1wy0L2yHvdaiqaacqWFZestdaahaaWcbeqaaiabd6eaobaaaaa@38E1@ (*s*, *t*) between a pair of iid random sequences with the same lengths as *s *and *t*, and with the probabilities of symbols set to the frequencies observed over *s *and *t*. This choice needs to have informations about the probability distributions of the optimal scores of *N*-maps. Even if the problem could sound more homogeneous than the alignment case, we failed to derive an analytical approximation of this distribution. However, two cases are quite simple to check:

*N *= 1 A 1-map is nothing but a gapless alignment of *s *and *t*. The distributions of the maximal scores were well studied in the local case and are known to converge to extreme value (EV) distributions [12].

*N *= |*s*| The optimal score of a |*s*|-map is obtained by summing the maximal substitution scores of all the positions of *s *against the whole sequence *t*. Let *t *be fixed and *s *be an iid sequence, then the scores associated to all the positions of *s *correspond to a set of iid random variables of expected value μt=∑x∈Apxmax⁡1≤p≤|t|{π[x,tp]}
 MathType@MTEF@5@5@+=feaafiart1ev1aaatCvAUfKttLearuWrP9MDH5MBPbIqV92AaeXatLxBI9gBaebbnrfifHhDYfgasaacH8akY=wiFfYdH8Gipec8Eeeu0xXdbba9frFj0=OqFfea0dXdd9vqai=hGuQ8kuc9pgc9s8qqaq=dirpe0xb9q8qiLsFr0=vr0=vr0dc8meaabaqaciaacaGaaeqabaqabeGadaaakeaaiiGacqWF8oqBdaWgaaWcbaGaemiDaqhabeaakiabg2da9maaqababaGaeeiCaa3aaSbaaSqaaGqabiab+Hha4bqabaGccyGGTbqBcqGGHbqycqGG4baEaSqaaiab+Hha4jabgIGioprtHrhAL1wy0L2yHvtyaeHbnfgDOvwBHrxAJfwnaGabaiab9bq8bbqab0GaeyyeIuoakmaaBaaaleaacqaIXaqmcqGHKjYOcqWGWbaCcqGHKjYOcqGG8baFcqWG0baDcqGG8baFaeqaaOGaei4EaSNae8hWda3aaSbaaSqaaiabcUfaBjab+Hha4jabcYcaSiabdsha0naaBaaameaacqWGWbaCaeqaaSGaeiyxa0fabeaakiabc2ha9baa@5FEC@ and variance σt2=∑x∈Apx(max⁡1≤p≤|t|{π[x,tp]}−μt)2
 MathType@MTEF@5@5@+=feaafiart1ev1aaatCvAUfKttLearuWrP9MDH5MBPbIqV92AaeXatLxBI9gBaebbnrfifHhDYfgasaacH8akY=wiFfYdH8Gipec8Eeeu0xXdbba9frFj0=OqFfea0dXdd9vqai=hGuQ8kuc9pgc9s8qqaq=dirpe0xb9q8qiLsFr0=vr0=vr0dc8meaabaqaciaacaGaaeqabaqabeGadaaakeaaiiGacqWFdpWCdaqhaaWcbaGaemiDaqhabaGaeGOmaidaaOGaeyypa0ZaaabeaeaaieaacqGFWbaCdaWgaaWcbaacbeGae0hEaGhabeaakiabcIcaOiGbc2gaTjabcggaHjabcIha4naaBaaaleaacqaIXaqmcqGHKjYOcqWGWbaCcqGHKjYOcqGG8baFcqWG0baDcqGG8baFaeqaaOGaei4EaSNae8hWda3aaSbaaSqaaiabcUfaBjab9Hha4jabcYcaSiabdsha0naaBaaameaacqWGWbaCaeqaaSGaeiyxa0fabeaaaeaacqqF4baEcqGHiiIZt0uy0HwzTfgDPnwy1egaryqtHrhAL1wy0L2yHvdaiqaacqaFaeFqaeqaniabggHiLdGccqGG9bqFcqGHsislcqWF8oqBdaWgaaWcbaGaemiDaqhabeaakiabcMcaPmaaCaaaleqabaGaeGOmaidaaaaa@67EF@, where p_x _is the probability of the symbol **x **in *s*. The optimal score turns out to be a sum of |*s*| random variables of this type. Thanks to the Central Limit Theorem, its distribution converges with |*s*| to the normal distribution N(μt|s|,σt2|s|)
 MathType@MTEF@5@5@+=feaafiart1ev1aaatCvAUfKttLearuWrP9MDH5MBPbIqV92AaeXatLxBI9gBaebbnrfifHhDYfgasaacH8akY=wiFfYdH8Gipec8Eeeu0xXdbba9frFj0=OqFfea0dXdd9vqai=hGuQ8kuc9pgc9s8qqaq=dirpe0xb9q8qiLsFr0=vr0=vr0dc8meaabaqaciaacaGaaeqabaqabeGadaaakeaat0uy0HwzTfgDPnwy1egaryqtHrhAL1wy0L2yHvdaiqaacqWFneVtcqGGOaakiiGacqGF8oqBdaWgaaWcbaGaemiDaqhabeaakiabcYha8jabdohaZjabcYha8jabcYcaSiab+n8aZnaaDaaaleaacqWG0baDaeaacqaIYaGmaaGccqGG8baFcqWGZbWCcqGG8baFcqGGPaqkaaa@4B64@. If *t *is not fixed but random, this distribution becomes a mixture of N(μt|s|,σt2|s|)
 MathType@MTEF@5@5@+=feaafiart1ev1aaatCvAUfKttLearuWrP9MDH5MBPbIqV92AaeXatLxBI9gBaebbnrfifHhDYfgasaacH8akY=wiFfYdH8Gipec8Eeeu0xXdbba9frFj0=OqFfea0dXdd9vqai=hGuQ8kuc9pgc9s8qqaq=dirpe0xb9q8qiLsFr0=vr0=vr0dc8meaabaqaciaacaGaaeqabaqabeGadaaakeaat0uy0HwzTfgDPnwy1egaryqtHrhAL1wy0L2yHvdaiqaacqWFneVtcqGGOaakiiGacqGF8oqBdaWgaaWcbaGaemiDaqhabeaakiabcYha8jabdohaZjabcYha8jabcYcaSiab+n8aZnaaDaaaleaacqWG0baDaeaacqaIYaGmaaGccqGG8baFcqWGZbWCcqGG8baFcqGGPaqkaaa@4B64@ with weights depending on the probabilities of sequences *t*. With reasonable assumptions about the length and the probability distribution of *t*, we can neglect all the components of the mixture except the one which has distribution N
 MathType@MTEF@5@5@+=feaafiart1ev1aaatCvAUfKttLearuWrP9MDH5MBPbIqV92AaeXatLxBI9gBaebbnrfifHhDYfgasaacH8akY=wiFfYdH8Gipec8Eeeu0xXdbba9frFj0=OqFfea0dXdd9vqai=hGuQ8kuc9pgc9s8qqaq=dirpe0xb9q8qiLsFr0=vr0=vr0dc8meaabaqaciaacaGaaeqabaqabeGadaaakeaat0uy0HwzTfgDPnwy1egaryqtHrhAL1wy0L2yHvdaiqaacqWFneVtaaa@383A@ (*μ*|*s*|, *σ*^2 ^|*s*|) where μ=∑x∈Apxmax⁡y∈A{π[x,y]}
 MathType@MTEF@5@5@+=feaafiart1ev1aaatCvAUfKttLearuWrP9MDH5MBPbIqV92AaeXatLxBI9gBaebbnrfifHhDYfgasaacH8akY=wiFfYdH8Gipec8Eeeu0xXdbba9frFj0=OqFfea0dXdd9vqai=hGuQ8kuc9pgc9s8qqaq=dirpe0xb9q8qiLsFr0=vr0=vr0dc8meaabaqaciaacaGaaeqabaqabeGadaaakeaaiiGacqWF8oqBcqGH9aqpdaaeqaqaaiabbchaWnaaBaaaleaaieqacqGF4baEaeqaaOGagiyBa0MaeiyyaeMaeiiEaG3aaSbaaSqaaiab+Lha5jabgIGioprtHrhAL1wy0L2yHvtyaeHbnfgDOvwBHrxAJfwnaGabaiab9bq8bbqabaGccqGG7bWEcqWFapaCdaWgaaWcbaGaei4waSLae4hEaGNaeiilaWIae4xEaKNaeiyxa0fabeaakiabc2ha9bWcbaGae4hEaGNaeyicI4Sae0haXheabeqdcqGHris5aaaa@571A@ and σ2=∑x∈Apx(max⁡y∈A{π[x,y]}−μ)2
 MathType@MTEF@5@5@+=feaafiart1ev1aaatCvAUfKttLearuWrP9MDH5MBPbIqV92AaeXatLxBI9gBaebbnrfifHhDYfgasaacH8akY=wiFfYdH8Gipec8Eeeu0xXdbba9frFj0=OqFfea0dXdd9vqai=hGuQ8kuc9pgc9s8qqaq=dirpe0xb9q8qiLsFr0=vr0=vr0dc8meaabaqaciaacaGaaeqabaqabeGadaaakeaaiiGacqWFdpWCdaahaaWcbeqaaiabikdaYaaakiabg2da9maaqababaGaeeiCaa3aaSbaaSqaaGqabiab+Hha4bqabaGccqGGOaakcyGGTbqBcqGGHbqycqGG4baEdaWgaaWcbaGae4xEaKNaeyicI48enfgDOvwBHrxAJfwnHbqeg0uy0HwzTfgDPnwy1aaceaGae0haXheabeaakiabcUha7jab=b8aWnaaBaaaleaacqGGBbWwcqGF4baEcqGGSaalcqGF5bqEcqGGDbqxaeqaaOGaeiyFa0NaeyOeI0Iae8hVd0MaeiykaKYaaWbaaSqabeaacqaIYaGmaaaabaGae4hEaGNaeyicI4Sae0haXheabeqdcqGHris5aaaa@5DB4@.

**Figure 3:**
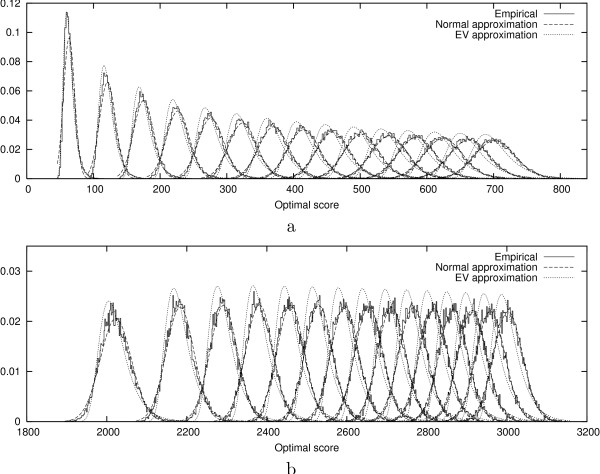
Empirical and approximated density functions of the optimal scores of local (a) and global (b) *N*-maps (*N *= 1, ..., 15 from left to right). The *N*-maps are computed using BLOSUM62 substitution matrix (made positive by adding a constant term in the global case) over 15000 random sequences with the same lengths and symbol distributions as Case study 5.2.

**Figure 4:**
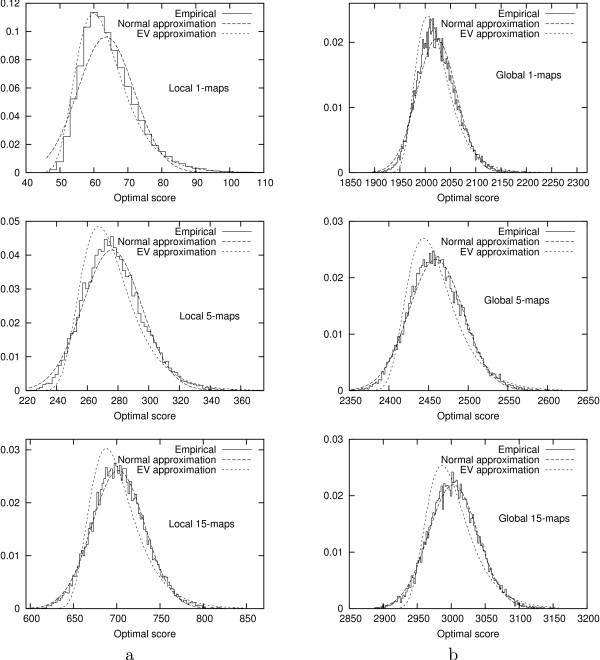
Empirical and approximated density functions of the optimal scores of: a) local 1-maps, 5-maps and 15-maps, b) global 1-maps, 5-maps and 15-maps. The *N*-maps are computed using BLOSUM62 substitution matrix over 15000 random sequences with the same lengths and symbol frequencies as Case study 5.2.

Figure 3 shows the evolution of the empirical density functions of optimal scores of *N*-maps with *N *in the range of 1 to 15. In Figure 4, we can see that the empirical density function corresponding to *N *= 1 in the local case is well approximated by an extreme value distribution. As *N *increases, even for small values, the empirical distributions differ more and more from extreme value distributions and approach quickly normal distributions both in local and global cases. For a given *N*, the empirical distributions of global optimal scores are closer to the normal approximations than the ones of local optimal scores.

Even if the distribution of optimal scores is of unknown form for intermediate values of *N*, the empirical observations show that normal approximations fit well except for very small values. This fact leads us to measure significance of the score of an optimal *N*-map in terms of *Z*-values as in [9]. The estimated *Z*-value of an optimal score ℳN
 MathType@MTEF@5@5@+=feaafiart1ev1aaatCvAUfKttLearuWrP9MDH5MBPbIqV92AaeXatLxBI9gBaebbnrfifHhDYfgasaacH8akY=wiFfYdH8Gipec8Eeeu0xXdbba9frFj0=OqFfea0dXdd9vqai=hGuQ8kuc9pgc9s8qqaq=dirpe0xb9q8qiLsFr0=vr0=vr0dc8meaabaqaciaacaGaaeqabaqabeGadaaakeaat0uy0HwzTfgDPnwy1egaryqtHrhAL1wy0L2yHvdaiqaacqWFZestdaahaaWcbeqaaiabd6eaobaaaaa@38E1@ (*s*, *t*) is the number of standard deviations separating this score from the mean:

Z(ℳN(s,t))=ℳN(s,t)−μ^N(s,t)σ^N(s,t)
 MathType@MTEF@5@5@+=feaafiart1ev1aaatCvAUfKttLearuWrP9MDH5MBPbIqV92AaeXatLxBI9gBaebbnrfifHhDYfgasaacH8akY=wiFfYdH8Gipec8Eeeu0xXdbba9frFj0=OqFfea0dXdd9vqai=hGuQ8kuc9pgc9s8qqaq=dirpe0xb9q8qiLsFr0=vr0=vr0dc8meaabaqaciaacaGaaeqabaqabeGadaaakeaacqWGAbGwcqGGOaakt0uy0HwzTfgDPnwy1egaryqtHrhAL1wy0L2yHvdaiqaacqWFZestdaahaaWcbeqaaiabd6eaobaakiabcIcaOiabdohaZjabcYcaSiabdsha0jabcMcaPiabcMcaPiabg2da9maalaaabaGae83mH00aaWbaaSqabeaacqWGobGtaaGccqGGOaakcqWGZbWCcqGGSaalcqWG0baDcqGGPaqkcqGHsisliiGacuGF8oqBgaqcamaaCaaaleqabaGaemOta4eaaOGaeiikaGIaem4CamNaeiilaWIaemiDaqNaeiykaKcabaGaf43WdmNbaKaadaahaaWcbeqaaiabd6eaobaakiabcIcaOiabdohaZjabcYcaSiabdsha0jabcMcaPaaaaaa@5C78@

where μ^N(s,t)
 MathType@MTEF@5@5@+=feaafiart1ev1aaatCvAUfKttLearuWrP9MDH5MBPbIqV92AaeXatLxBI9gBaebbnrfifHhDYfgasaacH8akY=wiFfYdH8Gipec8Eeeu0xXdbba9frFj0=OqFfea0dXdd9vqai=hGuQ8kuc9pgc9s8qqaq=dirpe0xb9q8qiLsFr0=vr0=vr0dc8meaabaqaciaacaGaaeqabaqabeGadaaakeaaiiGacuWF8oqBgaqcamaaCaaaleqabaGaemOta4eaaOGaeiikaGIaem4CamNaeiilaWIaemiDaqNaeiykaKcaaa@3547@ and σ^N(s,t)
 MathType@MTEF@5@5@+=feaafiart1ev1aaatCvAUfKttLearuWrP9MDH5MBPbIqV92AaeXatLxBI9gBaebbnrfifHhDYfgasaacH8akY=wiFfYdH8Gipec8Eeeu0xXdbba9frFj0=OqFfea0dXdd9vqai=hGuQ8kuc9pgc9s8qqaq=dirpe0xb9q8qiLsFr0=vr0=vr0dc8meaabaqaciaacaGaaeqabaqabeGadaaakeaaiiGacuWFdpWCgaqcamaaCaaaleqabaGaemOta4eaaOGaeiikaGIaem4CamNaeiilaWIaemiDaqNaeiykaKcaaa@3554@ denote respectively the mean and the standard deviation of the optimal scores estimated from a given number of trials of pairs of random sequences with the same lengths and the same frequencies of symbols as *s *and *t*. The higher the *Z*-value of ℳN
 MathType@MTEF@5@5@+=feaafiart1ev1aaatCvAUfKttLearuWrP9MDH5MBPbIqV92AaeXatLxBI9gBaebbnrfifHhDYfgasaacH8akY=wiFfYdH8Gipec8Eeeu0xXdbba9frFj0=OqFfea0dXdd9vqai=hGuQ8kuc9pgc9s8qqaq=dirpe0xb9q8qiLsFr0=vr0=vr0dc8meaabaqaciaacaGaaeqabaqabeGadaaakeaat0uy0HwzTfgDPnwy1egaryqtHrhAL1wy0L2yHvdaiqaacqWFZestdaahaaWcbeqaaiabd6eaobaaaaa@38E1@ (*s*, *t*), the lower the probability of observing a greater score in the normal approximation. So an optimal *N*-map of *s *over *t *with a higher *Z*-value will be consider more significant.

The *Z*-values must be taken with caution for small *N *– let us say less than 5 – because the corresponding probabilities are underestimated (the tails of the empirical distributions are heavier than the tails of the normal ones when *N *is smaller). This point is not crucial because we use *Z*-values to select a relevant number of part *N *rather than to assess an absolute significance of *N*-maps, but it could cause an underestimation of the "real" optimal number of parts. When the estimated most significant number of parts is small, it may be useful to check one or two next values.

Because it is time consuming and its accuracy is not rigorously evaluated, the way of estimating the significance of an optimal score is not fully satisfying. Analytical approximations of the distributions of the optimal scores should be pretty much better but they are beyond the scope of this article.

## 4 Evaluation

To evaluate the ability of the approach to retrieve segments of sequences related by evolution, we apply a given number of evolutionary events (mutations and shuffles) to random sequences and we measure the intersection between the homologies known from the artificial evolution and the ones reported by the most significant *N*-map.

More precisely, given a length *L*, a number of parts *K *and an identity proportion *α*, the protocol follows the steps below for a fixed number of trials.

1. Generate a reference random sequence *s*_*a *_iid with uniform probabilities over symbols of length *L *over an alphabet of 4 or 20 symbols ("random DNA" or "random protein").

2. Split *s*_*a *_into *K *equal parts and let *s*_*b *_be the sequence obtained by shuffling these parts with respect to the reverse permutation: (1, 2, ..., *K*) → (*K, K *- 1, ..., 1). This step defines a reference *K*-map of *s*_*a *_over *s*_*b*_.

3. Let s′b
 MathType@MTEF@5@5@+=feaafiart1ev1aaatCvAUfKttLearuWrP9MDH5MBPbIqV92AaeXatLxBI9gBaebbnrfifHhDYfgasaacH8akY=wiFfYdH8Gipec8Eeeu0xXdbba9frFj0=OqFfea0dXdd9vqai=hGuQ8kuc9pgc9s8qqaq=dirpe0xb9q8qiLsFr0=vr0=vr0dc8meaabaqaciaacaGaaeqabaqabeGadaaakeaacuWGZbWCgaqbamaaBaaaleaacqWGIbGyaeqaaaaa@2FA0@ be a sequence obtained by mutating (1 - *α*) × *L *different positions of *s*_*b *_randomly chosen with uniform probabilities. Here "mutating" implies an actual (and random) change of symbol, so the identity proportion between *s*_*b *_and s′b
 MathType@MTEF@5@5@+=feaafiart1ev1aaatCvAUfKttLearuWrP9MDH5MBPbIqV92AaeXatLxBI9gBaebbnrfifHhDYfgasaacH8akY=wiFfYdH8Gipec8Eeeu0xXdbba9frFj0=OqFfea0dXdd9vqai=hGuQ8kuc9pgc9s8qqaq=dirpe0xb9q8qiLsFr0=vr0=vr0dc8meaabaqaciaacaGaaeqabaqabeGadaaakeaacuWGZbWCgaqbamaaBaaaleaacqWGIbGyaeqaaaaa@2FA0@ is *α*. The *K*-map of Step 2 is kept as reference when mapping *s*_*a *_over s′b
 MathType@MTEF@5@5@+=feaafiart1ev1aaatCvAUfKttLearuWrP9MDH5MBPbIqV92AaeXatLxBI9gBaebbnrfifHhDYfgasaacH8akY=wiFfYdH8Gipec8Eeeu0xXdbba9frFj0=OqFfea0dXdd9vqai=hGuQ8kuc9pgc9s8qqaq=dirpe0xb9q8qiLsFr0=vr0=vr0dc8meaabaqaciaacaGaaeqabaqabeGadaaakeaacuWGZbWCgaqbamaaBaaaleaacqWGIbGyaeqaaaaa@2FA0@.

4. Determine the number *M *leading to the moat significant global *M*-map of *s*_*a *_over s′b
 MathType@MTEF@5@5@+=feaafiart1ev1aaatCvAUfKttLearuWrP9MDH5MBPbIqV92AaeXatLxBI9gBaebbnrfifHhDYfgasaacH8akY=wiFfYdH8Gipec8Eeeu0xXdbba9frFj0=OqFfea0dXdd9vqai=hGuQ8kuc9pgc9s8qqaq=dirpe0xb9q8qiLsFr0=vr0=vr0dc8meaabaqaciaacaGaaeqabaqabeGadaaakeaacuWGZbWCgaqbamaaBaaaleaacqWGIbGyaeqaaaaa@2FA0@ (by using Identity substitution matrix and by checking the *Z*-values for *M *between 1 and *K *+ 10).

5. Compute an optimal global *M*-map of *s*_*a *_over s′b
 MathType@MTEF@5@5@+=feaafiart1ev1aaatCvAUfKttLearuWrP9MDH5MBPbIqV92AaeXatLxBI9gBaebbnrfifHhDYfgasaacH8akY=wiFfYdH8Gipec8Eeeu0xXdbba9frFj0=OqFfea0dXdd9vqai=hGuQ8kuc9pgc9s8qqaq=dirpe0xb9q8qiLsFr0=vr0=vr0dc8meaabaqaciaacaGaaeqabaqabeGadaaakeaacuWGZbWCgaqbamaaBaaaleaacqWGIbGyaeqaaaaa@2FA0@ and measure its intersection with the reference *K*-map of Step 2, *i.e*. the number of pairs of positions of *s*_*a *_and s′b
 MathType@MTEF@5@5@+=feaafiart1ev1aaatCvAUfKttLearuWrP9MDH5MBPbIqV92AaeXatLxBI9gBaebbnrfifHhDYfgasaacH8akY=wiFfYdH8Gipec8Eeeu0xXdbba9frFj0=OqFfea0dXdd9vqai=hGuQ8kuc9pgc9s8qqaq=dirpe0xb9q8qiLsFr0=vr0=vr0dc8meaabaqaciaacaGaaeqabaqabeGadaaakeaacuWGZbWCgaqbamaaBaaaleaacqWGIbGyaeqaaaaa@2FA0@ which are associated both in a diagonal of the reference *K*-map and in a diagonal of the optimal *M*-map computed. Normalize this value by dividing by *L *= |*s*_*a*_| = |s′b
 MathType@MTEF@5@5@+=feaafiart1ev1aaatCvAUfKttLearuWrP9MDH5MBPbIqV92AaeXatLxBI9gBaebbnrfifHhDYfgasaacH8akY=wiFfYdH8Gipec8Eeeu0xXdbba9frFj0=OqFfea0dXdd9vqai=hGuQ8kuc9pgc9s8qqaq=dirpe0xb9q8qiLsFr0=vr0=vr0dc8meaabaqaciaacaGaaeqabaqabeGadaaakeaacuWGZbWCgaqbamaaBaaaleaacqWGIbGyaeqaaaaa@2FA0@| to get the *intersection ratio*.

We do not apply insertion/deletion events over sequences in the protocol because the approach deals with this type of evolution exactly in the same way as the "split and shuffle" of Step 2.

**Figure 5:**
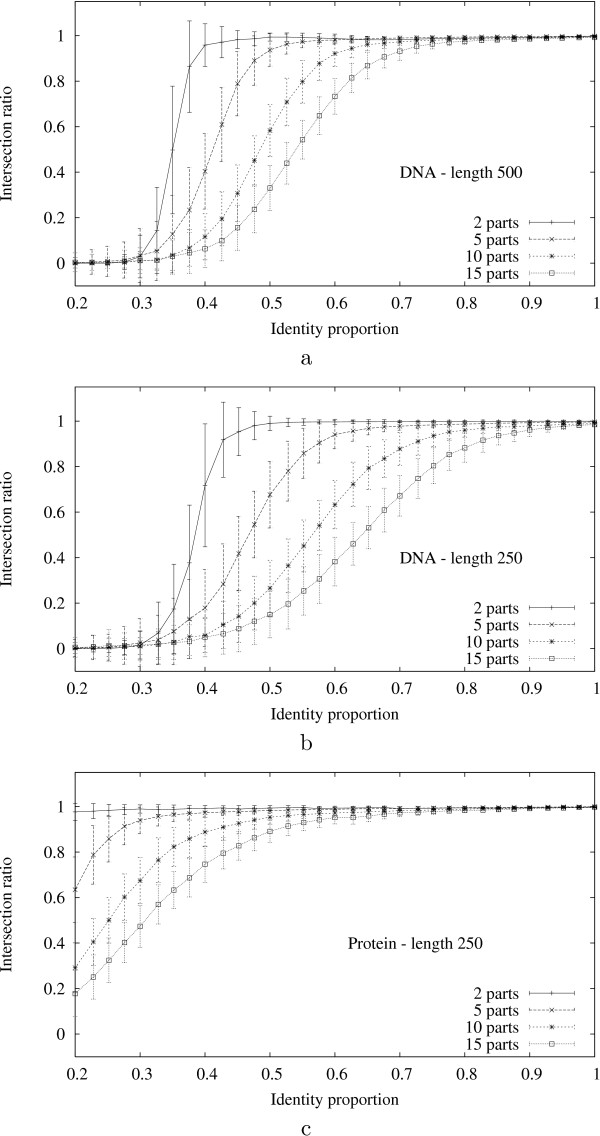
Evolution of the intersection ratio with the identity proportion for: a) random DNA sequences of length 500, b) random DNA sequences of length 250, c) random protein sequences of length 250.

Figure 5 shows the evolution of the means of the intersection ratios for *K *= 2, 5, 10 and 15 parts, as functions of the identity proportion conserved in Step 3, over:

• 500 random DNA sequences of length 500 (Figure 5a),

• 500 random DNA sequences of length 250 (Figure 5b),

• 500 random protein sequences of length 250 (Figure 5c).

The error bars displayed in Figure 5 report the corresponding standard deviations.

The agreement of the results is perfect or almost perfect when the identity proportion is high. The identity proportion, the number of parts, the length of the sequences and the number of symbols in the alphabet affect the intersection ratio. This can be explained by the fact that the ability of the approach to associate a given segment of *s*_*a *_with its artificially evolved counterparts in s′b
 MathType@MTEF@5@5@+=feaafiart1ev1aaatCvAUfKttLearuWrP9MDH5MBPbIqV92AaeXatLxBI9gBaebbnrfifHhDYfgasaacH8akY=wiFfYdH8Gipec8Eeeu0xXdbba9frFj0=OqFfea0dXdd9vqai=hGuQ8kuc9pgc9s8qqaq=dirpe0xb9q8qiLsFr0=vr0=vr0dc8meaabaqaciaacaGaaeqabaqabeGadaaakeaacuWGZbWCgaqbamaaBaaaleaacqWGIbGyaeqaaaaa@2FA0@ depends on the probability of observing another segment in s′b
 MathType@MTEF@5@5@+=feaafiart1ev1aaatCvAUfKttLearuWrP9MDH5MBPbIqV92AaeXatLxBI9gBaebbnrfifHhDYfgasaacH8akY=wiFfYdH8Gipec8Eeeu0xXdbba9frFj0=OqFfea0dXdd9vqai=hGuQ8kuc9pgc9s8qqaq=dirpe0xb9q8qiLsFr0=vr0=vr0dc8meaabaqaciaacaGaaeqabaqabeGadaaakeaacuWGZbWCgaqbamaaBaaaleaacqWGIbGyaeqaaaaa@2FA0@ with a better score (here identity proportion). For instance in the case of DNA sequences the expected identity proportion of two segments is 0.25 under the random model used in the protocol. So it is not surprising to observe that the intersection ratio is 0 when the identity proportion artificially required in Step 3 is smaller than this value (Figure 5a and Figure 5b). In the case of protein sequences, the expected identity proportion is 0.05. Eve tunes smaller than in the DNA case and we observe better results for small values of the identity proportion in Figure 5c. Clearly the identity proportion and the length of the sequence s′b
 MathType@MTEF@5@5@+=feaafiart1ev1aaatCvAUfKttLearuWrP9MDH5MBPbIqV92AaeXatLxBI9gBaebbnrfifHhDYfgasaacH8akY=wiFfYdH8Gipec8Eeeu0xXdbba9frFj0=OqFfea0dXdd9vqai=hGuQ8kuc9pgc9s8qqaq=dirpe0xb9q8qiLsFr0=vr0=vr0dc8meaabaqaciaacaGaaeqabaqabeGadaaakeaacuWGZbWCgaqbamaaBaaaleaacqWGIbGyaeqaaaaa@2FA0@ affect the probability of associating with the artificial counterpart. The role played by the number of parts is twofold. First, since it determines the length of the segments split in Step 2 of our protocol, it has a direct effect on the preceding probability. Second, it increases the number of boundaries and the possibility of an error when associating positions which are located at the beginning or at the end of the segments.

## 5 Case studies

**Figure 6:**
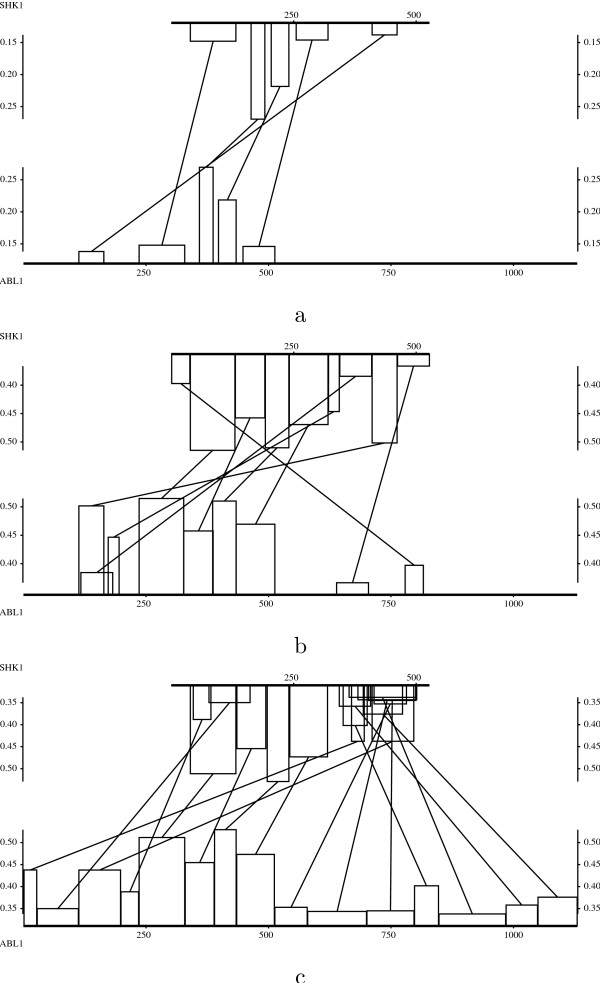
a) An optimal local 5-map both of SHK1 over ABL1 and of ABL1 over SHK1. b) An optimal global 9-map of SHK1 over ABL1. c) An optimal global 15-map of ABL1 over SHK1. Local and global maps are computed using BLOSUM62 substitution matrix (made positive by adding a constant term in the global case).

**Figure 7:**
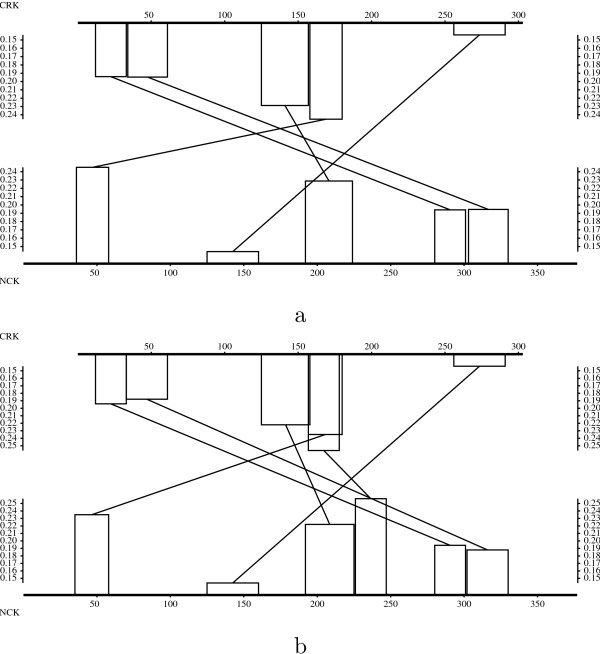
a) An optimal local 5-map of CRK over NCK. b) An optimal local 6-map of NCK over CRK. The *N*-maps are computed using BLOSUM62 substitution matrix.

**Figure 8:**
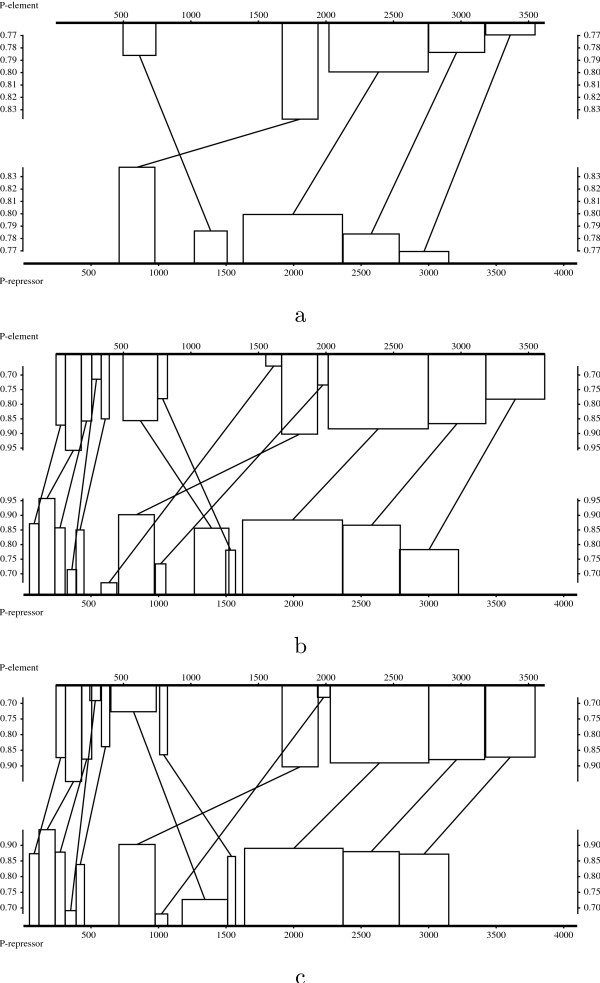
a) An optimal local 5-map of P-element over P-repressor and reciprocally (BLAST substitution matrix). b) An optimal global 15-map of P-element over P-repressor (Identity substitution matrix). c) An optimal global 24-map of P-repressor over P-element (Identity substitution matrix). Diagonals with average score (here identity proportion) smaller than 0.6 were removed from the global *N*-maps.

In the three first case studies, *N*-maps are represented as pictures where horizontal bold lines represent the sequences compared. The names of the sequences are specified over and under the lines. Each diagonal is represented as two boxes connected by an edge, where each box corresponds to a segment of one of the sequences. The height of the two boxes depends upon the score of the diagonal divided by its length (see Figures 6, 7 and 8). This type of graphical representation is also used in [1,6]. In the last case study we display *N*-maps as dotplots in order to make the results easily comparable with the ones of [13]. For convenience reasons, the scores represented in all the figures are normalized by being divided by the greatest entry of the substitution matrix.

### 5.1 Proteins 1

We begin with a case study from [3]. It compares SHK1 protein present in *Dictyostelium *(SwissProt ID Q9BI25) with ABL1 protein present in human (SwissProt ID ABL1_HUMAN). These proteins share two common domains which occur in a different order in each protein.

When comparing these sequences, the most significant optimal scores are obtained for:

• *N *= 5 for both local maps of SHK1 over ABL1 and of ABL1 over SHK1 (*Z*-values respectively 36.67 and 36.79 – Figure 6a),

• *N *= 9 for global map of SHK1 over ABL1 (*Z*-value 19.46 – Figure 6b),

• *N *= 15 for global map of ABL1 over SHK1 (*Z*-value 10.79 – Figure 6c).

With the global approach, each part of the sequence to map is associated to the segment which maximizes its gapless alignment score in the second sequence, even if this score is small and does not correspond to a "real" homology. As a result, a most significant global *N*-maps contain a greater number of diagonals and look more confusing than with the local case. However, despite the fact that not all the homologies reported are relevant, the global *N*-maps are interesting because they provide a more complete representation of the common elements. They allow us to consider diagonals formed by segments which are not homologous or not long enough to be selected in an optimal local *N*-map but which can be meaningful and can suggest an evolutionary history when taken in the whole context. Let us illustrate this point with the 15-map of ABL1 over SHK1 (Figure 6c). The diagonal with the ABL1-interval [1, 27] (the first segment of ABL1 in Figure 6c) is too short to be selected in the most significant optimal local *N*-map but it can make sense when taking into account the larger diagonal with the ABL1-interval [113,198] (the third segment of ABL1 in Figure 6c) that follows it – not consecutively – in the two sequences. This could suggest the deletion or the insertion of the ABL1-interval [28 – 112] along the evolutionary history of this protein.

A simple solution to make a global *N*-map clearer is to select only diagonals with scores greater than a given threshold and/or long enough (see Figure 8 – case study 5.3). In particular, by considering only diagonals with average scores over a threshold in the two global *N*-maps of this case study, we would obtain pictures very similar to the local 5-map.

Finally, the common domains reported in [3] are both retrieved in the homologies pointed out with the local and the global *N*-maps: Pkinase domain (positions about 110–200 in ABL1) and SH2 domain (positions about 230–510 in ABL1). The homology involving the SH2 domain is split into 4 diagonals in all the maps. Naturally, as these two domains are shuffled between the two sequences, a classical alignment could not point out the two homologies at once.

### 5.2 Proteins 2

We compare two proteins sequences from [6]. A CRK like protein (SwissProt ID P46109) and a NCK adaptor protein (SwissProt ID P16333). This example is given to illustrate the way of pointing out repeated elements and we consider only local *N*-maps. The most significant optimal scores are obtained for:

• *N *= 5 for local *N*-maps of CRK over NCK (*Z*-value 17.29 – Figure 7a),

• *N *= 6 for local *N*-maps of NCK over CRK (*Z*-value 18.91 – Figure 7b).

In Figure 7 we can see that the most significant optimal local *N*-map of NCK over CRK has an extra diagonal with regard to the *N*-map of CRK over NCK. Apart from this extra diagonal, these two maps share almost the same diagonals set. There are only some small changes on their boundaries, essentially because the non-overlapping constraint of Definition 1 applies either to one or the other sequence. The extra diagonal is composed of a segment of NCK (positions 226–247), which was not included in the diagonals of the reciprocal map, and a segment of CRK which is also part of another diagonal formed with the positions 35–58 of NCK. Since they are both homologous to a same segment of CRK, we have a clue that these two segments of NCK are repeated elements. Note that retrieving all the repeated common elements of two sequences needs generally to map one sequence over another and reciprocally to make sure that all the associations of segments are reported.

### 5.3 Transposons

We consider here DNA sequences of two transposons elements occurring in two species of *Drosophila *and studied in [14]: P-element (GenBank ID AY116625.1) and P-repressor (GenBank ID AF169142.2). We use the BLAST substitution matrix for nucleotides [15] for local *N*-maps and Identity matrix for global ones.

The most significant optimal scores of *N*-maps are obtained for:

• *N *= 5 for local maps both of P-element over P-repressor (*Z*-value 425.83) and of P-repressor over P-element (*Z*-value 420.24) corresponding to a same optimal 5-map (Figure 8a),

• *N *= 19 for global maps of P-element over P-repressor (*Z*-value 132.75 – Figure 8b),

• *N *= 24 for global maps of P-repressor over P-element (*Z*-value 119.96 – Figure 8c).

The corresponding maps are represented in Figure 8 in which we keep only the diagonals of the global *N*-maps with more than 60% of identity. As expected, filtering the diagonals according to their scores makes the pictures clearer and closer to the local one.

Once more, many diagonals are shared between these three *N*-maps with small variations in their-boundaries. The two global maps show an extra homologous region formed by several diagonals probably too short to be taken into account in the most significant local *N*-map.

In Figures 6, 7, and 8 we can remark series of diagonals composed of intervals of positions which seem contiguous and occur in the same order in the two sequences. They cannot be replaced by a unique diagonal because they are separated by small gaps (too small to appear at the scale of figures). In other words, *N*-maps computing acts over these positions like a classical alignment.

### 5.4 Microbial genomes

This case study illustrates how the approach can be applied to comparative genomics. We compare two microbial genomes: *Chlamydia trachomatis *(GenBank ID AE001273) and *Chlamydophila pneumoniae *(GenBank ID AE001363) studied in [13].

Each genome is represented by the sequence of its coding genes in the order they occur. Genomes of *Chlamydia trachomatis *and *Chlamydophila pneumoniae *contain respectively 895 and 1052 genes. A gene is identified with the sequence of amino acids of the corresponding protein. Thus, there are as much different symbols as the total length of the two genomes (except the unlikely case where several genes share exactly the same sequence of amino acids).

We compare two sequences/genomes *s *and *t *of symbols/genes which are themselves sequences of amino acids and we need to define a substitution score *π *between genes (actually this is only required between the genes of the first genome and the genes of the second one). For two sequences of amino acids *p*_*a *_and *p*_*b*_, we set *π*[*p*_*a*_, *p*_*b*_] to the (highest) identity proportion of an alignment of *p*_*a *_and *p*_*b*_. As this substitution score is non-negative, we will consider global *N*-maps.

**Figure 9:**
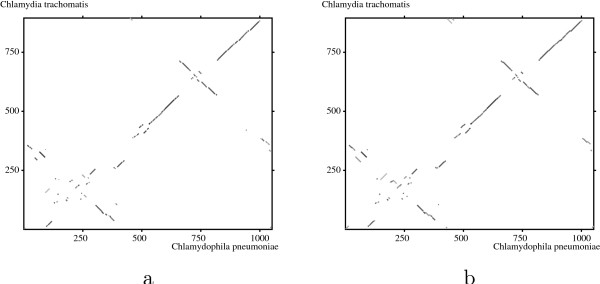
a) Dotplot representation of an optimal global 94-map of *Chlamydia trachomatis *over *Chlamydophila pneumoniae*. b) Dotplot representation of an optimal global 97-map of *Chlamydophila pneumoniae *over *Chlamydia trachomatis*. Darker is a diagonal, higher is its length-normalized score.

Because of the particular type of sequences studied here, the estimations of the empirical means and of the standard deviations of the *Z*-values are computed in a slightly different way from the one described in Section 3. To estimate the significance of a *N*-map score of a genome *s *over a genome *t*, we compute over a given number of trials, the empirical mean and the standard deviation of the optimal scores obtained by mapping a random shuffle of *s*, over *t*. The empirical distributions of the optimal scores observed by shuffling the first genome depend a lot on the nature of the substitution scores between the genes of the first genome and the genes of the second one. But in non degenerated cases (when the substitution levels between genes are not all the same) we observe a behaviour close to the one described in Section 3. In this case study the most significant optimal scores of global *N*-maps are obtained for:

• *N *= 94 for the global map of *Chlamydia trachomatis *over *Chlamydophila pneumoniae *(*Z*-value 289.61 – Figure 9a),

• *N *= 97 for the global map of *Chlamydophila pneumoniae *over *Chlamydia trachomatis *(*Z*-value 265.23 – Figure 9b).

Because of the number of diagonals involved in the rearrangement, which is relatively complex and includes several inversions, we represent *N*-maps as dotplots (see Figure 9). The authors of [13] use this type of representation and show similar figures.

The *N*-map approach allows us to perform genomes comparison without the initial step of identification of clusters of orthologous genes which is generally a necessary (and sometimes a critical) stage before comparing genomes [16,17]. However, the *N*-map approach is different to methods such as sorting by reversals because it does not construct an evolutionary history (in the sense that it does not provide a sequence of evolutionary events transforming the genomes). It is rather a way to connect conserved segments and can be seen as an alternative to identify orthologous genes. The fact that two genes are associated in a *N*-map does not depend only on the level of homology between these genes, but also benefits from the levels of homology between their respective neighbourhoods.

## 6 Discussion and future work

Mapping sequences by parts is a simple and effective way to find out similarities between two sequences in the presence of evolutionary events that do not preserve their linear order. This first version was written in order to introduce the idea of "computational mapping of sequences" and needs some technical improvements and extensions such as dealing differently with the bounds of the parts which are mapped or distinguishing different costs for mutational events, to become more realistic from a biological point of view.

In the local case, the optimal *N*-map of *s *over *t *is close to the selection of the *N *gapless alignments with higher scores. So the results obtained with local *N*-maps should be generally close to the ones obtained by methods based on local (gapped or not) alignments [2,3,6]. The main difference stands in the non-overlapping constraint of Definition 1. From our point of view, the originality of the method actually makes sense with global *N*-maps. Strengths and weaknesses of global *versus *local *N*-maps are analogous to the alignment case. The local approach allows us to report only significant homologies. But a drawback is that the level of significance needs to be fixed *a priori*, generally by shifting the entries of the substitution matrix more or less negatively. On the other hand, with the global approach (a positive matrix), adding a same positive constant to all the entries of the substitution matrix leaves the resulting optimal *N*-maps unchanged. A first drawback is that even weak homologies are reported, but this is not a real problem since they can be easily filtered. A more serious concern is that a strong homology can be possibly diluted in a longer (but weaker) one.

The method can be extended in several directions. A first natural way is to allow gaps while mapping each part of the first sequence. Basically it can be done by extending the definition of diagonal to not constrain the lengths of the two segments to be equal and by defining the score of an "extended diagonal" as the alignment score (penalizing gaps) of its two segments. The algorithms computing the maximal scores and optimal *N*-maps with extended diagonal scores (computed with linear or affine gap penalties) are essentially the same as **Alg_1 **and **Alg_2**. In particular, their orders of time and memory space complexities do not change. In fact, the current implementation of the method provides an option to align parts with a linear gap penalty. Nevertheless, we presented here the method with the gapless case because it appears conceptually clearer and does not need any parameter such as a gap penalty (this parameter is critical for the distributions of the optimal scores and they appear more confusing in the gapped case).

Further in the same direction, an interesting possibility of extension is to associate different kinds of penalties for insertions/deletions, inversions and shuffling, and to compute the greatest score of a map of *s *over *t *according to a substitution matrix and these penalties. From an algorithmic point of view and with reasonable kinds of penalties, this can be done by Dynamic Programming equations analogous to the ones used in **Alg_1**. These equations could be directly applied to compute the best score and an optimal set of diagonals of a "penalized map" of *s *over *t *with complexity *O *(|*s*| × |*t*|). Setting the different values of penalties is a natural way to introduce biological knowledges in the approach but this needs a strong expertise in sequence analysis. We are interested in collaborations in this direction.
